# CaSSiDI: novel single-cell “Cluster Similarity Scoring and Distinction Index” reveals critical functions for PirB and context-dependent Cebpb repression

**DOI:** 10.1038/s41418-024-01268-8

**Published:** 2024-02-21

**Authors:** Robert Nechanitzky, Parameswaran Ramachandran, Duygu Nechanitzky, Wanda Y. Li, Andrew C. Wakeham, Jillian Haight, Mary E. Saunders, Slava Epelman, Tak W. Mak

**Affiliations:** 1 Princess Margaret Cancer Centre, Ontario Cancer Institute, University Health Network, Toronto, ON Canada; 2 Centre for Oncology and Immunology, Hong Kong Science Park, Hong Kong SAR, China; 3 Toronto General Hospital Research Institute, University Health Network, Toronto, ON Canada; 4 Ted Rogers Centre for Heart Research, Translational Biology and Engineering Program, Toronto, ON Canada; 5 Peter Munk Cardiac Centre, UHN, Toronto, ON Canada; 6 Department of Laboratory Medicine and Pathobiology, University of Toronto, Toronto, ON Canada; 7 Departments of Immunology and Medical Biophysics, University of Toronto, Toronto, ON Canada; 8 Department of Pathology Department of Pathology, School of Clinical Medicine, Li Ka Shing Faculty of Medicine, The University of Hong Kong, Hong Kong SAR, China; 9 Present Address: Providence Therapeutics Holdings Inc., Calgary, AB Canada

**Keywords:** Immunology, Molecular biology

## Abstract

PirB is an inhibitory cell surface receptor particularly prominent on myeloid cells. PirB curtails the phenotypes of activated macrophages during inflammation or tumorigenesis, but its functions in macrophage homeostasis are obscure. To elucidate PirB-related functions in macrophages at steady-state, we generated and compared single-cell RNA-sequencing (scRNAseq) datasets obtained from myeloid cell subsets of wild type (WT) and PirB-deficient knockout (PirB KO) mice. To facilitate this analysis, we developed a novel approach to clustering parameter optimization called “Cluster Similarity Scoring and Distinction Index” (CaSSiDI). We demonstrate that CaSSiDI is an adaptable computational framework that facilitates tandem analysis of two scRNAseq datasets by optimizing clustering parameters. We further show that CaSSiDI offers more advantages than a standard Seurat analysis because it allows direct comparison of two or more independently clustered datasets, thereby alleviating the need for batch-correction while identifying the most similar and different clusters. Using CaSSiDI, we found that PirB is a novel regulator of *Cebpb* expression that controls the generation of Ly6C^lo^ patrolling monocytes and the expansion properties of peritoneal macrophages. PirB’s effect on Cebpb is tissue-specific since it was not observed in splenic red pulp macrophages (RPMs). However, CaSSiDI revealed a segregation of the WT RPM population into a CD68^lo^*Irf8*^+^ “neuronal-primed” subset and an CD68^hi^*Ftl1*^*+*^ “iron-loaded” subset. Our results establish the utility of CaSSiDI for single-cell assay analyses and the determination of optimal clustering parameters. Our application of CaSSiDI in this study has revealed previously unknown roles for PirB in myeloid cell populations. In particular, we have discovered homeostatic functions for PirB that are related to *Cebpb* expression in distinct macrophage subsets.

## Introduction

The murine “paired immunoglobulin-like receptor” (Pir)A and PirB molecules are expressed on osteocytes and leukocytes (especially myeloid cells), and transduce activatory and inhibitory signals through “immunoreceptor tyrosine-based activation motifs” (ITAMs) and the corresponding inhibitory motifs (ITIMs), respectively [1–3]. PirB is also expressed on neurons, where it blocks excessive axonal growth and neuronal regeneration [4]. Murine chromosome 7 carries six *PirA* genes and one *PirB* gene. The human orthologs of these genes are referred to as “leukocyte immunoglobulin-like receptors” (*LILR*) and are organized into the activatory LILRA family (*LILRA1–6*) and the inhibitory LILRB family (*LILRB1–5*).

Pir/LILR-mediated signaling modulates immune responses, and PirB-deficient knockout (PirB KO) mice develop exacerbated graft-versus-host disease [5]. Although the best characterized PirB ligand is the β2m component of MHC class I, other ligands have been implicated in PirB/LILRB-mediated responses, including non-classical MHC class I molecules such as Qa2 and Cd1d [6], as well as non-MHC molecules such as alarmins and pathogen epitopes [2, 3]. In cancer tissues, abnormal PirB/LILRB expression is linked to enhanced tumor growth and poor patient prognosis [7]. However, MHC class I-LILRB3 interactions can induce the extrusion of precancerous epithelial cells [8]. Therefore, it is unclear precisely how PirB shapes myeloid cell responses.

**Fig. 1:**
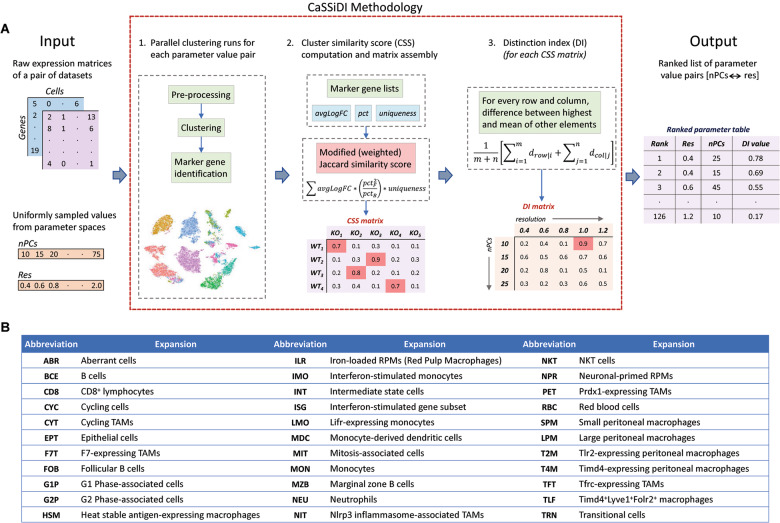
CaSSiDI framework and cluster identities. **A** Inputs: a pair of raw gene expression matrices for datasets whose clusters are to be matched, and uniformly sampled values from the parameter spaces to be optimized. **1**. Pre-processing, clustering, and marker gene identification steps for all parameter value pairs are conducted in parallel. **2**. For each parameter value pair, an $m\times n$ cluster similarity score (CSS) matrix is assembled containing scores for all possible cluster pairs between the two datasets using modified (weighted) Jaccard similarity. **3**. Finally, a single score for each CSS matrix, called the distinction index (DI), is computed in two steps. Row- and column-level DIs are computed as the differences between the row-wise and column-wise maximums and means. The mean across these $m+n$ DIs yields the matrix level DI. The final output is a ranked list of parameter value pairs from best to worst. **B** A listing of the 3-letter codes used throughout this paper to identify cell subsets and/or clusters, followed by their definitions.

Single-cell RNA sequencing (scRNAseq) assays provide valuable insights into cellular heterogeneities and functions, but the wide range of parameter choices (such as clustering resolution, number of reduced dimensions) can preclude accurate elucidation of novel activation states hidden within a cluster. In addition, most scRNAseq-based studies either do not describe the systematic methodology used to choose optimal parameter values such as clustering resolution or do not employ one at all. While deep-learning and AI-based approaches can identify novel cellular activation states [9–13], not all researchers have access to advanced bioinformatics analysis pipelines. Here, based on the Jaccard index and known features of cell lineages, we present a data-driven, semi-automated parameter optimization method called “Cluster Similarity Scoring and Distinction Index” (CaSSiDI) (Fig. 1A). Use of CaSSiDI narrows down the clustering-related parameter values to a manageable set of top choices that correspond to meaningful clustering conditions. In the process of developing CaSSiDI, we also constructed useful visualizations such as the “Nebula plot”, which illustrates (in a single plot) the top shared and differentially expressed genes (DEGs) and their expression levels for a given cluster pair within the context of average background expression. In this study, we use these tools to explore the role of PirB in diverse myeloid cell subsets and analyze its effects on their gene expression patterns. We present results on normal macrophage populations in our main text, and additional findings on tumor-associated macrophages (TAMs) in our Supplemental Results.

## Results

### CaSSiDI reveals heterogeneity and Cebpb derepression in PirB^−/−^Ly6C^+^ bone marrow-derived monocytes

**Fig. 2:**
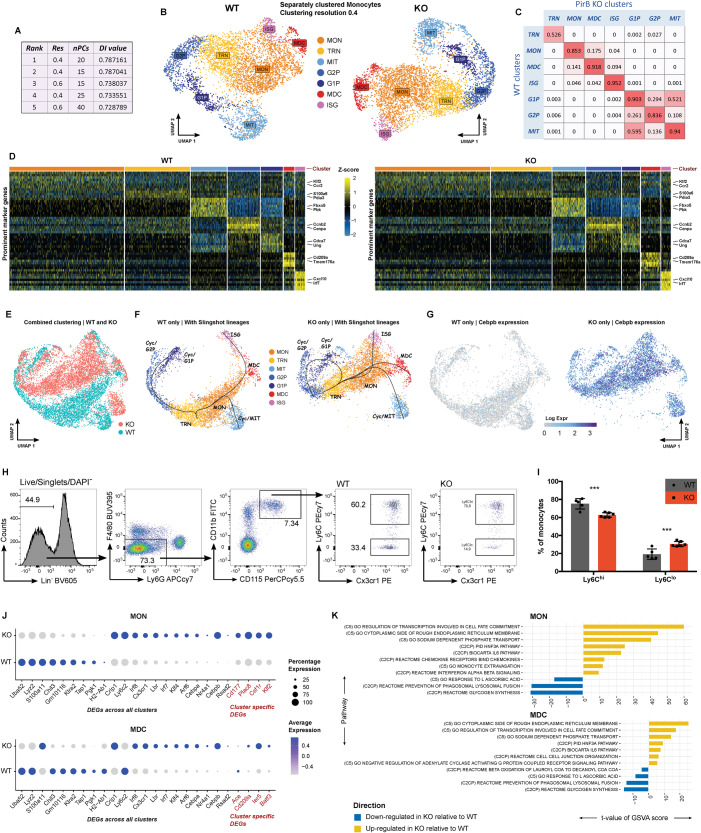
Steady-state monocytes: CaSSiDI reveals plausible WT and PirB KO clusters. **A** Top five parameter value pair choices as determined by CaSSiDI (*Res*: clustering resolution, *nPCs*: number of principal components). The top choice of res = 0.4, nPCs = 20 was used to cluster the WT and PirB KO datasets. **B** UMAP of separately clustered WT and PirB KO BM-derived monocytes. Please see Fig. 1B for definitions of abbreviations. **C** CSS table corresponding to the top-ranking choice in **A**. **D** Prominent marker genes for the indicated (by color) clusters. **E** UMAP of the combined clustering run of the WT and PirB KO monocyte populations. **F** Slingshot trajectory curves originating from the *TRN* cluster for the separately clustered WT and PirB KO monocyte populations laid out on the combined UMAP space. **G** UMAPs showing *Cebpb* expression for the WT and PirB KO monocyte populations in **F**. **H** Left: Flow cytometric gating strategy to identify blood-resident monocytes. Right: Results of this strategy applied to WT and PirB KO blood monocytes. **I** Quantitation of Ly6C^hi^ and Ly6C^lo^ blood monocytes in the WT and PirB-KO samples in **H** expressed as the percentage of total blood monocytes; WT *n* = 5, KO *n* = 6. ***P < 0.001 as determined by regression analysis with two-way analysis of variance (ANOVA) followed by Sidak’s post hoc multiple comparison test. Data are representative of two independent experiments. **J** Bubble plots showing prominent monocyte-related DEGs (PirB-dependent in black; cluster-specific in red) between WT and PirB KO cells within the indicated clusters as determined by the CSS table in **C**. **K** Top differentially expressed pathways identified by GSVA between WT and PirB KO cells within the indicated clusters. Also shown are t-value scores from a linear model analysis conducted on GSVA scores.

To examine PirB’s role in monocyte cell subsets, we used flow cytometry to isolate Lin^-^CD11b^+^CD115^+^Ly6G^-^Ly6C^+^ bone marrow (BM)-derived monocytes from WT and *Pirb*^*−/−*^ (PirB KO) female littermate mice and subjected them immediately to scRNAseq. Our CaSSiDI approach (Fig. 1 and see Methods) determined that a resolution (res) of 0.4 and 20 principal components (nPCs) was the optimal set of parameter choices (hereafter, clustering condition) for comparing WT to PirB KO monocytes (Fig. 2A). Seven WT and PirB KO monocyte cluster pairs with high similarity were identified (Fig. 2B, C), with 3 out of 7 pairs representing cycling cells. [Fig. 1B shows a comprehensive legend for all 3-letter cluster identification codes used in this paper.] Examination of cluster-associated marker genes revealed cells expressing *Fbxo5*, *Pbk*, *Cdk1*, *Aurkb* (linked to mitosis; MIT) in both WT and mutant cultures, as well as cells expressing *Cdca7, Asf1b, Ung* (in G1 phase/S phase; G1P), and those expressing *Ccnb2, Birc5, Cenpa* (in G2 phase, G2P) (Fig. 2D; Supplementary Tables S1 and S2). Notably, the premonocytic marker *Cxcr4* [14] was expressed by WT and mutant cells in the MIT and G1P clusters (Supplementary Tables S1 and S2).

We sought to elucidate the heterogeneity within specific monocyte subsets from WT and PirB KO mice. In both WT and mutant animals, a cluster termed MON showed high mRNA expression of classical monocyte-associated marker genes, including *Ccr2* (monocyte egress from the BM) [15–17]; the transcription factor (TF) *Klf2* (inhibits proinflammatory monocyte activation) [18]; as well as *Plac8*, *Csf1r* and the classical neutrophil marker *Cd177* (Fig. 2D, J). The monocyte-derived dendritic cell (MDC) cluster showed high mRNA levels of the MHC class II molecules H2-Ab1, H2-Eb1, and H2-Dmb1, as well as the DC-related markers *Cd74*, *Cd209a*, *Batf3, Tmem176b* [19–21] (Fig. 2D, J) (Supplementary Tables S1 and S2). The monocyte subset expressing high mRNA levels of *Irf7*, *Ifit3, Cxcl10* was termed the interferon-stimulated gene (ISG) subset (Fig. 2D, Supplementary Fig. S1A). The transitional cell (TRN) subset showed only a handful of upregulated genes, including S100a6, a calcium-binding protein and critical regulator of myeloid output and mitochondrial metabolism in hematopoietic stem and progenitor cells [22]. However, most genes associated with the TRN subset were downregulated (Fig. 2D, Supplementary Fig. S1A; Supplementary Tables S1 and S2), which is a common feature of progenitor or transitory cell populations [23]. Notably, the PirB KO TRN subset expressed *Pdia3* (Supplementary Table S2), which facilitates hematopoietic progenitor cell anchorage in the BM [24].

The phenotype of the TRN cluster prompted us to use it as the starting point for a Slingshot analysis (Fig. 2E, F). As is typical for progenitor-like cells, the trajectories of the WT and mutant MON, MDC, and ISG subsets indicated that the TRN subset likely possesses self-renewal capacity, with one differentiation path confined to the cycling clusters G2P and G1P and another path leading to the MON state. From there, we observed the MIT state (likely MON amplification) and the emergence of MDC and ISG cells from MON cells. These trajectory-related findings were equivalent in the corresponding WT and PirB KO clusters.

One striking difference between almost all WT and PirB KO monocyte subsets was an increase in mRNA levels of the transcription factor *Cebpb* in the mutant cells (Fig. 2G). We surmised that PirB might be required for *Cebpb* repression in BM-derived monocytes, implicating PirB in regulating the differentiation of Ly6C^hi^ into Ly6C^lo^ monocytes as well as into monocyte-derived macrophages and DCs [25, 26]. Indeed, flow cytometric assessment of blood monocyte subsets revealed a higher proportion of Ly6C^lo^ patrolling monocytes at the expense of Ly6C^hi^ monocytes in PirB KO mice compared to WT animals (Fig. 2H, I).

Although all corresponding WT and mutant monocyte clusters were highly similar (Fig. 2C), an impartial DE analysis and volcano plotting revealed numerous differentially expressed genes (DEGs) (Supplementary Fig. S1B–E). We identified 21 monocytic core genes that were deregulated in the absence of PirB (Fig. 2J and Supplementary Fig. S1A, black gene designations), as well as four DEGs that differed in a cluster-specific manner (Fig. 2J and Supplementary Fig. S1A, red gene designations). Global pathway analysis revealed that PirB deficiency affected numerous pathways but particularly “Response to L-ascorbic acid” and “Regulation of transcription involved in cell fate commitment” (Fig. 2K and Supplementary Fig. S1F). Taken together, these data indicate that PirB restricts *Cebpb* expression in monocytes.

### Benchmark validation of CaSSiDI as an approach to improve clustering

The results above indicated that CaSSiDI determined an optimal clustering condition that reliably predicted a range of established monocyte subsets. To validate our CaSSiDI method, we compared our data outcomes to those produced using a Standard Seurat Pipeline (SSP) procedure for cluster determination (see Supplemental Results for a detailed description). The standard Seurat approach recommends using res = 0.8 and determining the number of PCs by elbow plots, plotting nPCs against the standard deviation [27]. Accordingly, the optimal nPC value can be chosen approximately where the curve takes an elbow turn (Fig. S1G). Our application of SSP to our data above assigned parameters of res=0.8 and nPC=10, generating nine WT and eight PirB KO monocyte clusters (Fig. S1H). Inspection of the marker genes associated with these clusters revealed the following insights, which highlight the utility of CaSSiDI. 1) For the WT sample, the MON, MDC, and the three cell cycle-associated clusters (G1P, G2P, MIT) showed similar arrays of marker genes when analyzed by SSP or CaSSiDI (Supplementary Table S3). However, SSP also assigned the largest cluster (ART) to cells that did not display positively regulated genes (Supplementary Table S3; Supplementary Fig. S1H). This outcome is not biologically meaningful and thus can only be interpreted as an artifact stemming from a suboptimal data analysis pipeline. 2) SSP assigned two clusters (TRN and TRN2) to cells containing only 41 or 12 positively regulated genes, respectively. These genes were predominantly linked to progenitor- and cell cycle-associated features, making it difficult to derive distinct biological functions (Supplementary Table S3). 3) The last two WT monocyte clusters (ISG and ISG2) identified by SSP shared a similar ISG-related gene expression signature that included *Irf7, Stat1, Oasl2, Ifi47*, which is likely also due to over-clustering (Supplementary Table S3). 4) For the PirB KO monocyte dataset, both SSP and CaSSiDI identified MON, MDC, ISG, G1P, G2P, and MIT clusters with similar gene signatures. However, SSP generated an additional cluster with a vast number of cell cycle-related genes (CYC), as well as two transitional cell clusters (TRN and TRN2) with fewer than 12 positively regulated marker genes each (Supplementary Table S4). These clusters were functionally hard to interpret, making the SSP-derived outcome less accurate and harder to work with than those obtained with CaSSiDI-determined clustering.

Our comparative analysis against SSP establishes that CaSSiDI-based determination of optimal clustering conditions simplifies the detailed comparison of two scRNAseq datasets, and produces biologically more relevant outcomes. We repeated this CaSSiDI versus SSP comparison for splenic macrophages and peritoneal macrophages and found the same improvement in clustering using the former over the latter (see below and Supplemental Results).

### CaSSiDI reveals two functional subsets of WT red pulp macrophages

**Fig. 3:**
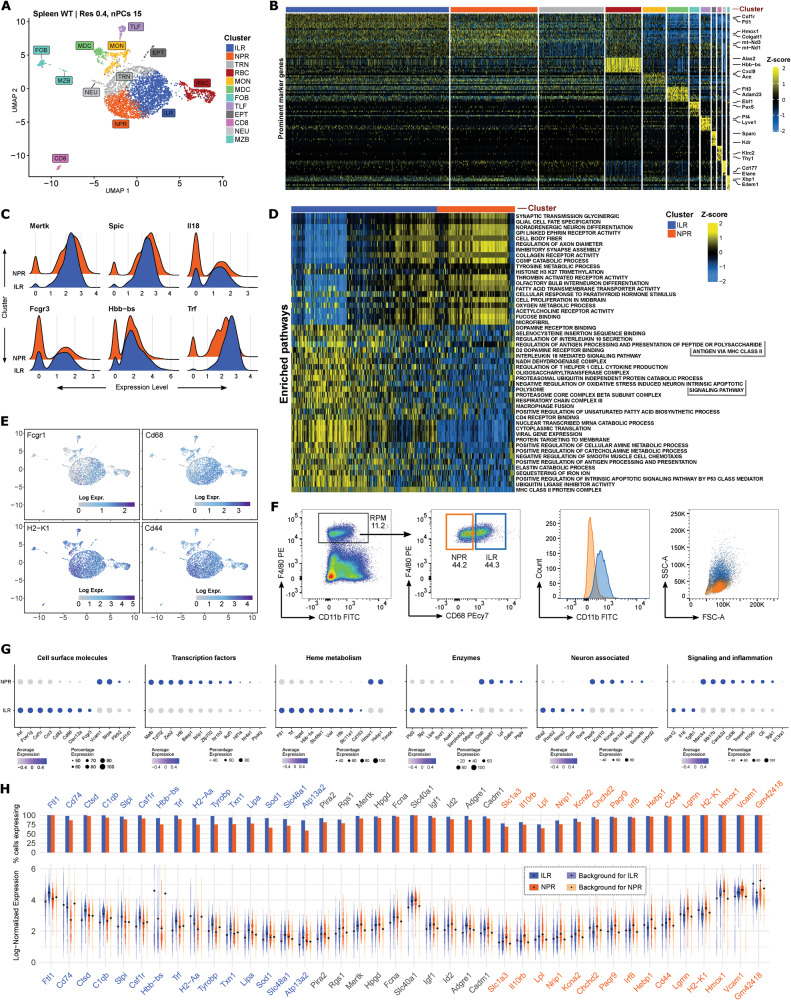
Spleen: WT NPR vs. WT ILR clusters. **A** UMAP for clusters of WT splenic red pulp macrophages (RPMs) obtained using the indicated top-ranked CSS parameter values. **B** Prominent marker genes for the clusters (indicated by color) in **A**. **C** Ridge plots showing expression levels of the indicated DEGs between WT ILRs (blue) and NPRs (orange). **D** Top differentially expressed pathways identified by GSVA between WT ILRs (blue) and NPRs (orange). **E** UMAPs showing expression levels of the indicated genes in WT splenic RPMs. **F** Left: Flow cytometric gating strategy to identify ILRs and NPRs among WT RPMs. CD3^+^, CD19^+^ and NK1.1^+^ cells were excluded (please see Methods). ILRs (blue) and NPRs (orange) were distinguished by their CD68 expression. Right: Flow cytometric analysis to show differences in CD11b expression and cell morphology between WT ILRs and NPRs. **G** Bubble plots organized by the indicated gene groups illustrating DEGs between WT ILRs and NPRs. **H** Nebula plot showing prominent ILR-specific (blue, left), shared (gray, middle), and NPR-specific (orange, right) marker genes. Please see Methods for a full explanation of this plot type.

Having established that CaSSiDI could reliably identify known subsets among BM-derived monocytes, we applied our approach to sorted splenic red pulp macrophages (RPMs) from WT mice. The primary function of RPMs is homeostatic scavenging of senescent erythrocytes and recycling of their heme and iron [28, 29], but RPMs may also control *P. falciparum* infections [28, 30]. It is unclear if these differing functions are executed by two distinct RPM subsets [31, 32]. We therefore isolated the Lin^-^F4/80^+^CD11b^int^MHCII^hi^ RPM population from digested spleens of WT female littermate mice. CaSSiDI determined that the most suitable clustering condition in this case was res = 0.4 and nPCs = 15 (Supplementary Fig. S2A). As expected, the steady-state WT RPM population showed contamination by other cell populations (Fig. 3A). Inspection of cluster-associated marker genes showed no over- or under-clustering, meaning that CaSSiDI yielded biologically meaningful clusters with a non-redundant distribution of marker genes. (Fig. 3B; Supplementary Table S5). A small macrophage cluster expressed *Lyve1*, *Folr2*, *Fcrls, Pf4*, likely representing a yolk sac-derived TLF subset [33]. However, the pan-macrophage marker *Mertk* [34, 35] and the bona fide RPM marker *Spi-C* [36, 37] were confined to two enormous clusters (orange and blue) in the UMAP plot (Fig. 3A; Supplementary Table S5). Another cluster (gray) showed elevated expression levels (as compared with other clusters) of only 16 genes, with 13 being associated with mitochondria (Fig. 3B). The remaining upregulated genes, namely *Lgmn, Siglece, Hebp1*, suggested that these cells were in a transitory state (TRN, Supplementary Table S5) [23].

Cluster and pathway analyses of the blue and orange clusters (ILR and NPR, respectively, abbreviations defined below) revealed two RPM subsets (Fig. 3A–E). Orange cluster cells expressed classical RPM genes like *Hmox1, Vcam1, Hepb1*. Because this cluster was linked to neuronal-associated pathways such as “Synaptic transmission glycinergic”, “Glial cell fate specification”, and “Noradrenergic neuron differentiation”, it was deemed to contain “Neuronal-primed RPMs” (NPRs). Blue cluster cells expressed a different set of classical RPM genes involved in either iron storage or antigen presentation, including *Csf1r, Ftl1, Ctsd, H2-Ab1, CD74* (Fig. 3A–E, G, Supplementary Fig. S2B). We termed the cells in this cluster “Iron-loaded RPMs” (ILRs). ILRs also showed greater expression of *Mertk, Spic, Fcgr3, Il18* as well as erythrocyte-associated hemoglobin (*Hbb-bs*) and serotransferrin (*Trf*) (Fig. 3C, G), suggesting that ILRs contain more iron than NPRs. The higher iron load and IL-18 expression in ILRs may better equip these cells to eliminate bacteria. Accordingly, GSVA pathway analysis of the blue cluster highlighted the global gene expression programs “MHC class II protein complex”, “Sequestering of iron ion”, and “Positive regulation of antigen processing and presentation” (Fig. 3D).

Next, we performed a COMET analysis [38] of WT RPMs to determine the most suitable cell surface antigen combinations for differential isolation of NPRs vs. ILRs (Fig. 3E). Unexpectedly, only CD68 showed a sufficient difference in expression to facilitate separation of ILRs and NPRs by flow cytometry (Supplementary Fig. S2C). Separating Lin^-^F4/80^+^CD11b^int^ into CD68^lo^ and CD68^hi^ cells (NPRs and ILRs, respectively) revealed a positive correlation between CD68 and CD11b expression (Fig. 3F). Thus, NPRs express low levels of CD68 and CD11b, whereas ILRs express elevated levels of both of these surface markers. Strikingly, this correlation was also reflected in cell morphology, with ILRs exhibiting increased cell size (FSC-A) and granularity (SSC-A) compared to NPRs (Fig. 3F). These differences may reflect a maturation process whereby macrophages that can engage in phagocytosis (NPRs) become those that can no longer do so (ILRs) and instead rely on iron-based mechanisms to combat pathogens.

Among DEGs (Fig. 3G), ILRs showed increased *Ftl1, Trf, Slc48a1, Hfe, Sod1*, which are involved in heme/iron biology [29, 39]. In contrast, the transcription factors *Zeb2, Irf, Ikzf1* were more highly expressed in NPRs than ILRs. *Zeb2* safeguards tissue-specific macrophage states, while *Irf8* increases macroautophagy [40], and *Ikzf1* is associated with an anti-inflammatory macrophage phenotype [41, 42].

To account for background gene expression derived from clusters outside a population of interest, we custom-designed a visualization that we call the “Nebula plot” and applied it to a comparison of NPRs and ILRs (Fig. 3H). Genes in blue are the top 15 ILR-related genes, followed by the top 10 genes shared between ILRs and NPRs in black, and the top 15 NPR-related genes in orange. Close to 100% of ILRs and NPRs expressed *Slc40a1* (ferroportin) [43] and *Adgre1* (F4/80). Other shared surface receptor genes were *Mertk* and *Pira2* (co-expressed with PirB) [34, 35]. Genes expressed primarily by ILRs included *Ftl1*, *Trf*, *Hbb-bs*. In NPRs, the most highly expressed genes were *Hmox1, Vcam1, GM42418*.

We sought to validate candidate genes identified by our scRNA-seq DEG analysis pipeline. To this end, we sorted WT splenic Lin^-^F4/80^+^CD11b^int^ macrophages into the CD68^lo^ and CD68^hi^ subsets, prepared cDNAs, and examined expression levels of selected candidate genes by qPCR (Supplementary Fig. S2D). As expected, there was no difference in *Slc40a1* transcript levels between ILRs and NPRs, confirming our original finding shown in Fig. 3H. ILRs showed increased *Ctsd* mRNA levels, whereas *Paqr9* and *Cd44* mRNAs were reduced in ILRs compared with NPRs (Supplementary Fig. S2D). *Hmox1* and *Vcam1* showed a trend towards decreased expression in ILRs compared to NPRs but these differences were not statistically significant.

Thus, our dissection of steady-state WT RPMs by CaSSiDI and the Nebula plot has revealed two subsets with substantial bona fide gene expression differences. NPRs display a neuronal-associated gene expression program and express high levels of *Hmox1* and *Hebp1* to recycle senescent erythrocytes. ILRs may be fully loaded with iron and therefore prepared to fight bacteria efficiently.

### PirB deficiency alters gene expression patterns and subset proportions among steady-state RPMs

**Fig. 4:**
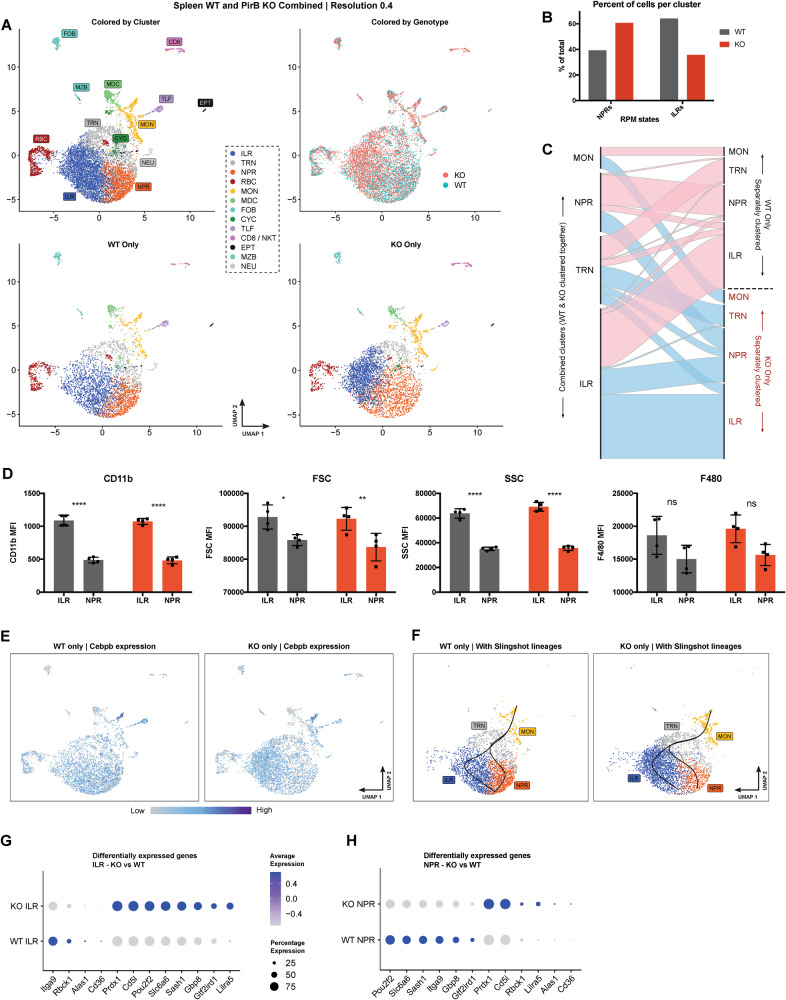
Spleen: WT NPR and ILR vs. PirB KO NPR and ILR clusters. **A** UMAPs showing combined clustering results of WT and PirB KO splenic RPMs, with cells grouped as indicated: by combined cluster identities, by genotype, and by identities obtained from clustering the WT and KO cells separately. **B** Quantitation of percentages of NPRs and ILRs among WT and PirB KO RPMs in **A**. **C** Alluvial diagram linking the combined clusters and the separate clusters, showing the flow of cells and the regroupings between the combined and separate clustering strategies. **D** Quantitation of the mean fluorescent intensities (MFI) of the indicated features discriminating between NPRs and ILRs isolated from spleens of WT and PirB KO mice (WT *n* = 4, KO *n* = 4) using the gating strategy shown in Fig. 3F. *P < 0.05, **P < 0.01, ****P < 0.0001 as determined by regression analysis with two-way ANOVA followed by Sidak’s post hoc multiple comparison test. Data are representative of two independent experiments. **E** UMAPs showing *Cebpb* mRNA levels in WT and PirB KO RPMs. **F** Slingshot trajectory curves originating from the *MON* cluster for the separately clustered WT and PirB KO RPM populations laid out on the combined UMAP space. **G**, **H** Bubble plots comparing prominent DEGs in WT vs. PirB KO ILRs and WT vs. PirB KO NPRs.

To dissect PirB’s functions in RPMs, we compared RPM samples from WT and PirB KO female littermate mice using a combined clustering approach and detected the same ILR and NPR clusters as revealed by CaSSiDI-based separate clustering (Fig. 4A, Supplementary Fig. S3A, B). Visual inspection of UMAP plots suggested an increase in NPRs at the expense of ILRs in the mutant. Indeed, enumeration of WT and PirB KO cells per cluster confirmed this trend, suggesting a role for PirB in RPM differentiation or subset-specific proliferation (Fig. 4B). We next performed an alluvial plot analysis confirming the close match between the separate and combined clustering approaches (Fig. 4C). The greatest difference was observed in the TRN cluster, where most cells were correctly assigned to the separate PirB KO TRN cluster (457 cells), but another 231 and 173 cells were allocated to the separate PirB KO ILR and NRP clusters, respectively (Supplementary Table S6). Flow cytometric differences in CD11b expression, cell size (FSC-A), and granularity (SSC-A) differed to the same degree between ILRs and NPRs of WT and PirB KO mice. F4/80 expression was comparable across WT and mutant ILRs and NPRs (Fig. 4D). Strikingly, unlike PirB KO monocytes, PirB KO RPMs did not show elevated *Cebpb* (Fig. 4E).

A Slingshot trajectory analysis confirmed that both WT and PirB KO steady-state monocytes become TRN RPMs that differentiate into NPRs or ILRs (Fig. 4F). When we investigated DEGs in WT vs. PirB KO ILRs, we found that mutant ILRs showed drastic decreases in *Itga9*, *Rbck1*, *Alas1, Cd36*, whereas *Pou2f2, Slc6a6, Sash1, Gbp8, Gtf2ird1, Lilra5* were elevated in these cells (Fig. 4G, Supplementary Fig. S3C). In comparing WT and PirB KO NPRs, we found that *Pou2f2, Slc6a6, Sash1, Itga9, Gbp8, Gtf2ird1* transcripts were less abundant in PirB KO NPRs, but that *Rbck1, Lilra5, Alas1, CD36* mRNAs were all elevated (Fig. 4H, Supplementary Fig. S3D). *Prdx1* and *Cd5l* (inflammatory mediators) were significantly increased in both the ILRs and NPRs of the mutant compared to the WT (Fig. 4G, H). GSVA comparing WT vs. PirB KO NPRs and ILRs showed increased expression of pathways involving secretion and regulation of IL-10, IL-12, and IL-13 in both mutant subsets (Supplementary Fig. S3E). Thus, a lack of PirB impinges on global RPM and subset-specific gene expression patterns at steady-state.

Lastly, as noted above, we applied SSP to the above splenic macrophage dataset and obtained inferior clustering outcomes (Supplementary Fig. S8A, B; Supplemental Results). This result bolsters the validity of our CaSSiDI method.

### Validation of the CaSSiDI approach by analysis of Zeb2 in splenic myeloid cells

The TF *Zeb2* drives expression of essential RPM-related genes. To validate our CaSSiDI approach in a biological context, we chose a published study by Scott et al. that contained scRNAseq data obtained from steady-state WT and Zeb2 KO RPMs [44]. The combined clustering analysis carried out therein focused on WT vs. Zeb2 KO differences and did not pinpoint cluster-associated features or identify RPM subsets. We processed the Scott data through our CaSSiDI pipeline and compared the Scott results with data from our scRNAseq analysis of steady-state WT RPMs.

When we compared clusters of steady-state PirB WT and Zeb2 WT samples, both were found to contain the ILR and NPR RPM subsets (Supplementary Fig. S4A). The TRN cluster was absent from both WT populations, but a myeloid cell cluster with an ISG signature (*Cxcl9, Stat1, Ifit2, Marco, Ifi47, Fcgr4, Cd274, Icam1*) was present in both samples (Supplementary Fig. S4A, Supplementary Tables S7 and S8). Importantly, NPRs and ILRs were readily identified in the independent biological replicates represented by the PirB WT and Zeb2 WT samples, despite their differences in quality and quantity of contaminating non-macrophage cell types (Supplementary Fig. S4A, B). Thus, CaSSiDI operates in a manner analogous to data integration pipelines. Moreover, the top clustering condition chosen by CaSSiDI in Supplementary Fig. S4C (res = 0.4, nPCs = 35) differs from that in comparing PirB WT and PirB KO RPMs in Fig. 4 (res = 0.4, nPCs = 15). This outcome indicates that CaSSiDI is sensitive to the nature of the input data.

**Fig. 5:**
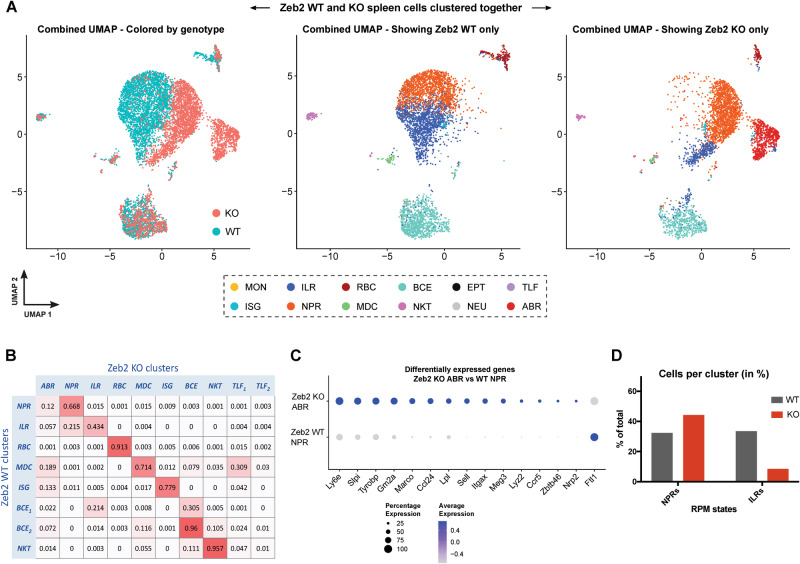
Validation of CaSSiDI utilizing scRNA-seq data from Zeb2 KO RPMs. **A** UMAPs showing combined clustering results of WT and Zeb2 KO RPMs, with cells grouped as indicated: by genotype, and by identities obtained from clustering the WT and KO samples separately. **B** CSS table comparing clusters obtained using CaSSiDI on data from splenic WT and Zeb2 KO samples presented in ref. [44]. **C** Bubble plots illustrating prominent DEGs between the Zeb2 WT NPR and Zeb2 KO ABR populations of ref. [44]. **D** Quantitation of percentages of NPRs and ILRs among the Zeb2 WT and Zeb2 KO RPM populations of ref. [44].

We then used CaSSiDI to compare steady-state Zeb2 WT and Zeb2 KO clusters. Once again, the ILR and NPR RPM subsets were identified, ISG cells appeared, and the TRN cluster was absent (Fig. 5A, B). Each WT RPM cluster was highly similar to the corresponding Zeb2 KO cluster (Fig. 5B), but a previously unidentified cluster (ABR*,* red) appeared in Zeb2 KO RPMs (Fig. 5A, B). In line with Zeb2’s control of classical DC development [45–47], these red cluster cells co-expressed genes typical of not only macrophages but also DCs and mast cells, in particular *Itgax* (CD11c), *Zbtb46*, *Ly6e, Slpi, Gm2a* (Fig. 5C, Supplementary Fig. S4D). Compared to PirB deficiency, loss of Zeb2 had a greater skewing effect on the proportions of ILR vs. NPR cells among RPMs (Fig. 5D). These results confirmed the vital function of Zeb2 in RPM subset differentiation and bolstered our confidence in our CaSSiDI method.

### PirB deficiency alters gene expression patterns of peritoneal macrophage subsets

RPMs, alveolar macrophages (AMs), and peritoneal macrophages (PMs) are tissue-resident macrophages (TRMs) that maintain their numbers by slow and local self-renewal [33]. In contrast, skin- and dermis-associated TRMs are constantly replaced by circulating monocytes [48, 49]. The factors controlling local self-renewal versus monocyte-dependent replacement are not fully understood but may include sex-dependent differences and elements of the tissue-specific microenvironment [50, 51].

**Fig. 6:**
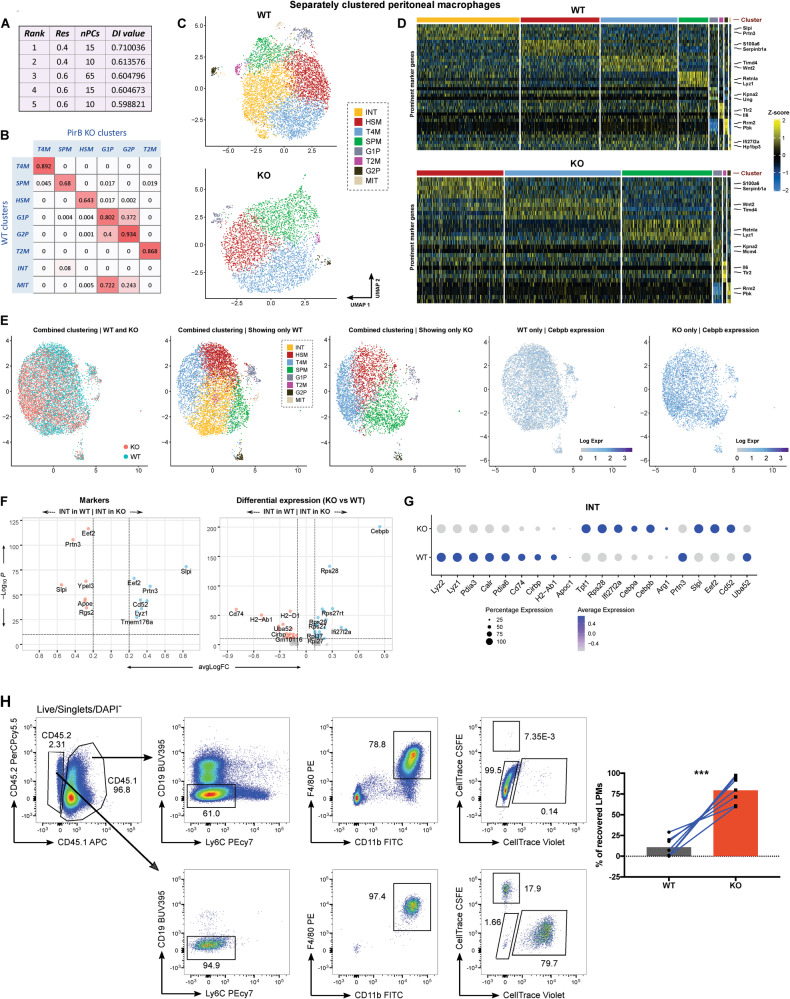
Peritoneum: WT vs. PirB KO PMs. **A** Top five parameter value pair choices as determined by CaSSiDI. The top choice of res = 0.4, nPCs = 15 was used to cluster the WT and PirB KO PM datasets. **B** CSS table corresponding to the top-ranking choice in **A**. **C** UMAPs of separately clustered WT and PirB KO PM populations. **D** Prominent marker genes for the indicated (by color) clusters. **E** UMAPs showing combined clustering results of WT and PirB KO PMs, with cells grouped as indicated: by genotype, and by identities obtained from clustering the WT and KO samples separately. The far-right plots are color-coded by *Cebpb* expression level. Volcano (**F**) and bubble (**G**) plots comparing markers and DEGs expressed by WT vs. PirB KO cells that occupied the INT cluster as identified using the combined clustering approach. **H** Left: Flow cytometric gating strategy for the identification of unlabeled CD45.1^+^ recipient LPMs or donor CD45.2^+^ LPMs, which were either labeled WT (CellTrace CSFE) or PirB KO (CellTrace Violet) LPMs. Right: Quantitation of WT and PirB KO LPMs recovered from CD45.1 recipients (WT *n* = 7, KO *n* = 7) at day 12 after co-transplantation. ***P < 0.001 as determined by two-tailed, paired t-test. Data are representative of two independent experiments.

There are two PM subsets: large PMs (LPMs) and small PMs (SPMs). SPMs are 10x less abundant than LPMs and express F4/80^lo^ (*Adgre1*), *Cd226, Retnla*. Irf4 is indispensable for SPM generation [50, 52, 53]. In contrast, LPM differentiation is governed by *Gata6, Cebpb*, retinoic acid, and omentum-derived factors [54, 55]. It is mainly LPMs that conduct immunosurveillance of the peritoneal cavity and adjacent viscera [56, 57]. To determine if PirB contributes to PM heterogeneity and function, we compared scRNAseq datasets from steady-state WT and PirB KO PMs. CaSSiDI established that the optimal clustering condition was res = 0.4 and nPCs = 15 (Fig. 6A), which yielded 8 WT and 6 PirB KO total PM cell clusters (Fig. 6B, C). Among WT PMs, we identified three clusters (G1P, G2P, and MIT) that each contained small numbers of cells showing an overrepresentation of cell cycle-related genes. Only the G1P and G2P clusters appeared in the KO PM population (Fig. 6B, D) (Supplementary Tables S9 and S10). It is possible that an MIT cluster was not detected due to the low cell numbers in that cluster and/or an ovelap in cell cycle genes with other clusters. Notably, PirB deficiency caused global derepression of *Cebpb* in PMs as it did in monocytes (Fig. 6E).

The WT PM population also contained a cluster (yellow) that likely represented an intermediate state (INT) and was lacking in the KO population (Fig. 6B–D). The INT cluster exhibited few upregulated genes but did include *Prtn3* (a neutrophil serine protease; NSP), which contributes to the control of LPM vs. SPM differentiation [54]. NSPs are repressed during the monocyte-macrophage transition [58]. *Prtn3* limits the self-renewal of hematopoietic progenitors [59] and may do the same for self-renewing LPMs. INT cells also expressed *Slpi*, an anti-apoptotic regulator that controls NSP activities and safeguards the granulocyte lineage [60–62]. Additional INT cluster-linked genes were *Ypel3* (p53 target) [63, 64]; *Rgs2* (negative regulator) [65]; and *Cirbp* (mediates hemorrhagic shock and sepsis) [66] (Fig. 6C, D; Supplementary Table S9). Combined clustering confirmed the absence of the INT cluster in PirB KO PMs (Fig. 6E). Notably, cells of the PirB KO SPM cluster (green), which expressed the SPM signature gene *Retnla* (Fig. 6D), spread into the region of the WT INT cluster (Fig. 6E). This observation suggests that the WT INT cluster is stabilized in a prospective SPM state; that is, it contains monocytes destined to undergo SPM differentiation. The absence of an equivalent INT cluster in the PirB KO PM population reinforces the reported effect of PirB deficiency on BM-derived monocytes, which rapidly adopt a macrophage-like state upon BM egress [67, 68]. Interestingly, our PirB KO SPMs expressed the WT INT gene *Slpi* (Fig. 6F, G, Supplementary Table S10).

Consistent with previous work [51], our CaSSiDI pipeline elucidated three subsets of WT mature functional LPMs: T2M (*Tlr2*-expressing macrophages), T4M (*Timd4*-expressing macrophages), and HSM (*Heat stable antigen (CD24)*-expressing macrophages). For details on the phenotypes of these subsets, please see Supplemental Results.

We next set out to test potential functional differences between WT and PirB KO PMs. Of note, *Cebpb* expression is known to be a prerequisite for LPM generation [55]. We adoptively transferred WT or PirB KO CD3^-^CD19^-^CD11c^-^Ly6C^lo^CD11b^hi^F4/80^hi^ LPMs (*CD45.2*^*+*^) into B6 recipient mice harboring the congenic marker *CD45.1*. Consistent with the elevated *Cebpb* mRNA levels observed in PirB KO PMs, we recovered greater than four-fold more CD45.2^+^, donor-derived PirB KO LPMs from recipients than WT LPMs at 21 days post-transplantation (Supplementary Fig. S5A). To overcome potential biases due to individual and/or host-dependent transplantation factors, we performed a competitive adoptive transfer experiment in which equal numbers of purified and dye-labeled CD45.2^+^ WT (CellTrace CSFE) and CD45.2^+^ PirB KO (CellTrace Violet) LPMs were co-transplanted into the same recipient mouse bearing unlabeled CD45.1^+^ WT LPMs. By 12 days after co-transplantation (the manufacturer-indicated guarantee of dye stability), significantly more CellTrace Violet^+^ PirB KO LPMs than CellTrace CSFE^+^ WT LPMs were recovered from recipients (Fig. 6H). These data indicate that *PirB*-deficient LPMs have a competitive edge over WT LPMs following adoptive transfer.

Taken together, these data identify PirB as a molecule that represses *Cebpb* expression to limit the differentiation or expansion of PMs in the peritoneal cavity. However, as was true for RPMs, loss of PirB has only mild effects on non-differentiating PM subsets at steady-state.

Finally, as noted above, we applied SSP to the above peritoneal macrophage dataset and obtained inferior clustering outcomes (Supplementary Fig. S8C, D; Supplemental Results). Once again, this result demonstrates the beneficial qualities of our CaSSiDI approach.

## Discussion

In this study, we have devised a novel single-cell cluster matching and optimization approach called CaSSiDI, and have used it to elucidate several cell type-associated differences in gene expression patterns between WT and PirB KO myeloid cell subsets. CaSSiDI proved to have several important advantages compared to a Standard Seurat Pipeline (SSP) in producing interpretable clustering outcomes. The objective of CaSSiDI is to find similar clusters in any two independently clustered scRNAseq datasets. To this end, CaSSiDI utilizes a marker gene-based quantitative score to carry out side-by-side comparisons of clusters across multiple resolutions and numbers of principal components in a grid-search fashion (See Methods for further details). Importantly, CaSSiDI considers only positive marker genes (genes overexpressed in a cluster compared to the background population) to determine cluster similarities. Negative markers (genes underexpressed in a cluster compared to the background population) were often found to be redundant across clusters and therefore unreliable for use in cluster identification. Moreover, CaSSiDI prevents the distortion of cell cluster allocations stemming from genetically divergent samples (e.g., WT vs. KO) that can occur when a classical combined clustering approach requiring batch correction is utilized. That being said, joint application of the separate and combined clustering approaches can help reveal biological relevance and correlations between two scRNAseq samples of interest. In this study, we have illustrated this utility by our analyses of murine RPMs, PMs, and TAMs (see Supplemental Results).

In addition to circumventing the need for batch correction, CaSSiDI’s rank and score tables highlight cluster similarities in a graded fashion and thus also indicate potential cluster trajectories in a single glance. Notably, we used CaSSiDI to straightforwardly identify known monocyte and PM subsets in our WT samples. Based on this success, we were able to use CaSSiDI to explore heterogeneities within TRM populations that are less well-described in the literature. Our data have thus expanded knowledge in this field.

CaSSiDI’s enhanced ability to refine complex single-cell data analysis should be useful in several scenarios. First, as demonstrated for the scRNAseq data in this study, CaSSiDI can be applied to optimize parameters for a combined clustering run by pitting the combined dataset (WT + KO) against the WT or KO samples individually. Depending on how much the WT and KO datasets differ, running CaSSiDI twice, once with WT and once with KO, may yield either the same best parameter value pair or two different choices. In the latter situation, the final decision on which of the two choices must be accepted for the combined clustering can be made by carefully considering the biological relevance and contexts associated with the clusters.

Second, CaSSiDI can effectively identify cell types in an uncharacterized dataset using a dataset for which the number and types of clusters have already been characterized. A CaSSiDI run with the characterized and uncharacterized datasets as inputs will systematically look for the best cluster matches at optimal resolution and nPCs and, in the process, characterize or label the clusters in the new dataset.

Third, CaSSiDI is not restricted to just RNAseq data or optimizing the resolution and nPCs parameters. It can be applied to data from other types of single-cell assays such as CYTOF and CITE-seq, and will work with any parameter pair that significantly influences clustering outcomes in a setting where one-to-one matches are expected to exist across cluster sets originating from a pair of datasets. The framework can also be easily expanded to include more than two parameters at a time for optimization, as long as computational resources are adequate. If, for example, we introduce a third parameter with 10 sampled values in addition to our usual set of res and nPCs, we would then have to run the module 1260 times, yielding as many CSS matrices. Since the runs are entirely independent, they can be done in parallel using a multicore computer cluster or a cloud-based computing service, both of which are ubiquitous and cheap to access at present. Once the clustering runs are complete and the CSS matrices are prepared, the DI computations are simple enough to execute on a standard laptop computer.

Fourth, depending on available computational resources, the number of samples from each parameter space can be increased to improve the power and accuracy of the search. For example, we have chosen our nPCs values to range from 10 to 75 in steps of 5, yielding 14 values. We can increase this to 33 values by reducing the step size to 3. Similarly, we can increase the resolution space by reducing the step size to 0.1 from 0.2. Consequently, many more parallel runs of the modules would be required. Nevertheless, since the runs are independent and can be executed in parallel, they should not consume any additional computational time if a sufficient number of parallel cores are available.

CaSSiDI does have a few limitations. First, CaSSiDI employs a grid-search style parameter optimization approach, which confines the method’s output to the predefined grid points and ranges for each parameter search. If the actual optimal values fall outside the specified ranges or reside between the grid values, CaSSiDI may yield near-approximations instead. This hurdle is a fundamental challenge associated with grid searches in general, and can be mitigated to some extent by widening the parameter ranges and employing denser search spaces (provided sufficient computational resources are available). Second, while CaSSiDI can hint at possible cluster trajectories, it is not designed as a primary trajectory inference method and should not be employed as such. It may overlook intricate trajectory structures and complex networks, which can only be unveiled through dedicated methods specifically designed for these purposes. Third, CaSSiDI is not an independent clustering method. Its performance relies on the quality of the initial clustering approach used to create clusters at different resolutions. Hence, selecting the most appropriate clustering method for the specific data type is paramount, as it forms the foundation upon which CaSSiDI operates effectively.

Turning to our biological results, perhaps this study’s most critical conclusion is our unexpected finding that PirB is important for repressing *Cebpb* in some myeloid cell subsets at steady-state. Cebpb induces the differentiation of classical Ly6C^hi^ monocytes [26] and is also required for proper macrophage differentiation in normal BM, peritoneal cavity, and lung [26, 55, 69]. However, this engagement appears to be tissue-specific since, although PirB KO PMs showed elevated *Cebpb* mRNA levels and a precocious monocyte-to-macrophage transition, PirB KO RPMs did not differ from the WT in their *Cebpb* abundance.

Another factor to consider is that the *Cebpb* mRNA encodes two activating TFs called “Liver-enriched activator proteins” (LAPs) and one repressive TF called “Liver-enriched inhibitory protein” (LIP); the expression of these TFs is known to be differentially regulated [68, 70]. Furthermore, the translation of LAPs vs. LIP may be context- and cell type-dependent, and also cross-regulated by other cell signaling pathways [71]. Thus, the differential and antagonistic functions of LAPs and LIP, as well as their multifaceted pre- and post-translational control, makes the dissection of *Cebpb* regulation challenging. We found that loss of PirB upregulated some genes in certain macrophage subsets while downregulating others, suggesting that signaling through PirB controls gene expression in ways other than through *Cebpb* repression. Additional study is required to resolve precisely how PirB-mediated signaling affects Cebpb-derived TFs during the generation of various myeloid cell subsets. These findings also highlight the need to elucidate the natural PirB ligands in various tissues and contexts that are required to invoke *Cebpb* expression and/or repression.

Lastly, our analyses have uncovered a previously unknown RPM differentiation/maturation process that generates NPRs and ILRs. These subsets differ in cell size, morphology, and surface expression of CD11b and CD68. Our scRNA-based analyses suggest that NPRs are *Irf8*^*+*^ and represent a macrophage state that is ‘ready to eat’, whereas ILRs are ‘done eating’, generate IL-18, and bear an increased iron content that may allow them an alternative means of combatting pathogens. Future studies will be needed to determine the factors that govern PM maturation.

In conclusion, we believe that our novel CaSSiDI method makes a valuable contribution to the field of single-cell assay analysis, and that we have established its utility by demonstrating a role for PirB in the repression of *Cebpb* in certain subsets of myeloid lineage cells.

## Methods

### Mice

PirB knockout mice (Pirb^−/−^ 129/B6 mice; [72]) were purchased from The Jackson Laboratory. Female littermates, age 11-13 weeks, were used for all scRNAseq experiments. All animals were maintained in fully accredited facilities at the Princess Margaret Cancer Centre. No particular means for animal randomization or blinding investigators were implemented during experimentation.

### Cell preparations, cell sorting, and flow cytometry

Cell preparations and flow cytometric analyses were performed essentially as described [73]. In brief, mice were humanely euthanized utilizing CO_2_ displacement and cervical dislocation. Bone marrow-derived monocytes were recovered by flushing the bone marrow of femur plus tibia with ice-cold FACS buffer (PBS^−/−^ supplemented with 2% heat-inactivated FCS and 2 mM EDTA). To obtain splenocyte single-cell suspensions, spleens were crushed through a 70 µm filter using a syringe and suspended in 4 ml ice-cold FACS buffer. For peritoneal lavage, 5-7 ml ice-cold FACS buffer was injected into the peritoneal cavity followed by abdominal massage. Buffer containing peritoneal cells was aspirated by syringe equipped with a 20 G needle. All cell suspensions were subjected to red blood cell (RBC) lysis using a commercially available buffer (Sigma). Cells were pelleted by centrifugation (300 × *g*, 5 min, 4 °C), resuspended in ice-cold FACS buffer, and subjected to cell count determinations.

All single-cell suspensions were treated with Fc-blocking antibody (anti-CD16/32; 2.4G2; Tonbo) before staining with titered antibody dilutions. The following antibodies were used for cell surface staining. Lin^-^ staining: anti-CD3 (145-2c11), anti-CD19 (1D3), anti-NK1.1 (PK136), anti-Ter119 (Ter119), anti-TCRgd (GL3). Target cell identification: anti-CD11b (M1/70), anti-CD11c (N418), anti-F4/80 (BM8), anti-CD115 (AFS98), anti-CD45.1 (A20), anti-CD45.2 (104), anti-CD68 (FA-11), anti-Ly6C (HK1.4), anti-Ly6G (1A8), anti-Cx3cr1 (SAO11F11), anti-MerTK (108928), anti-CD64 (X54-5/7.1). All antibodies were purchased from BD Biosciences, BioLegend, or Thermo Fisher Scientific. Dead cells were stained and excluded using 12.5 ng/ml DAPI (Sigma).

### Adoptive transfer of peritoneal macrophages

Peritoneal cells were harvested by lavage of the peritoneal cavity with 7 ml cold PBS. Cells were washed using FACS buffer (PBS with 2% heat-inactivated FBS) and passed through a 70 µm filter before being stained with the appropriate antibodies as indicated in the Results section, the Figures, and the Figure legends. LPMs were sorted as indicated using a BD FACSAria Fusion cell sorter. WT and PirB KO LPMs were counted and labeled with CellTrace CSFE or CellTrace Violet, respectively, according to the manufacturer’s instructions (Thermo Fisher Scientific). Equal numbers of purified and dye-labeled CD45.2^+^ WT and PirB KO LPMs were co-transplanted into the same recipient mouse bearing unlabeled CD45.1^+^ WT LPMs.

### Single-cell RNA sequencing

Processing of murine cells for scRNAseq was performed essentially as previously described [33]. In brief, BM monocytes, splenic macrophages, peritoneal macrophages, or B16 tumor cells were isolated by flow cytometric cell sorting at low pressure. Single-cell suspensions were prepared according to the Chromium Single Cell 3’ Reagent Kits User Guide (v2 Chemistry). Samples were loaded onto a 10x Chromium instrument to produce sequencing libraries, which were processed according to methods provided by 10x Genomics. Cells were sequenced and processed to generate expression matrices using Cell Ranger (10x Genomics). Raw base call (BCL) files from a HiSeq2500 sequencer were demultiplexed into FASTQ files, which were aligned (STAR) and filtered, followed by barcode and UMI counting to generate the counts table. The scRNAseq package Seurat v3.1.0 [74] was used for all downstream analyses using R 3.6.1. To take advantage of some improved plotting features, Seurat v3.2.3 in R v4.0.3 was also selectively used.

### CaSSiDI: cluster similarity scoring methodology

The rationale and derivation of CaSSiDI are presented in detail in Supplemental Methods. In brief, one of the main challenges we faced in analyzing our scRNAseq data was in arriving at the most meaningful set of values for the computational parameters that wield a significant influence on biological inference: the clustering resolution (res) and the number of principal components (nPC) that are input for clustering. We also had the unique requirement of comparing and contrasting the PirB KO datasets with their corresponding WT counterparts. Therefore, it was necessary to find a set of parameter values that, on the one hand, revealed as many direct one-to-one cluster mappings as possible between WT and KO (thus identifying the same or similar cell types/states), while on the other hand identified any stand-alone clusters that were unique to either the WT or KO population.

To achieve these objectives, we devised a multi-step strategy that: 1) sampled from the space defined by res and nPCs; 2) computed the clusters corresponding to each pair of parameter values chosen from the space; 3) evaluated clustering quality using a score; and 4) used the scores to determine the best pair of parameter values satisfying predefined criteria. The score characterizing clustering quality was designed such that it rewards parameter value pairs that identify clear one-to-one matches between WT and KO clusters along with any clusters unique to one genotype, but penalizes parameter value pairs that yield partial or unclear matches or too many clusters (over-clustering). In other words, we sought to find the parameter value pair that brought out the most striking one-to-one correspondences between WT and KO cluster sets while, at the same time, kept the number of clusters as small as possible to avoid over-clustering.

The first step in our strategy involves choosing the set of values for the res and the nPC parameters that will represent our sampled space of search. For all our analyses, we set nPC to range from 10 to 75 in steps of 5 and res to range from 0.4 to 2 in steps of 0.2. This translates into 14 nPC values and 9 res values, or a total of 126 value pairs. We ran Seurat’s clustering module for each of these pairs for both the WT and KO datasets and identified markers for the clusters. Only markers satisfying an adjusted p-value threshold of 0.05 were retained. These markers were then used to compute a cluster similarity score (CSS) between each WT cluster and KO cluster using a weighted and enhanced similarity metric derived from the popular Jaccard similarity coefficient (https://www.jstor.org/stable/2427226). For two clusters with marker gene sets M_1_ and M_2_, a CSS can be defined using the original Jaccard similarity coefficient as

$${{CSS}}_{{basic}}=\frac{{C}_{12}}{{C}_{12}+{U}_{1}+{U}_{2}}$$

where ${C}_{12}$ is the number of common markers between clusters 1 and 2, ${U}_{1}$ is the number of markers unique to cluster 1, and ${U}_{2}$ is the number of markers unique to cluster 2. The score ranges between 0 and 1, with 1 representing identical clusters with all markers being shared and 0 representing the most dissimilar clusters with no common marker genes. While the ${{CSS}}_{{basic}}$ formulation is an acceptable measure of similarity, it is too simple in this context as it is solely based on gene names and does not take into consideration other essential measurements such as the proportion of cells expressing each marker, the average expression strength, or how exclusively the gene serves as a marker for a cluster. All of these additional measurements are important indicators of the effectiveness of a marker gene for a cluster. We reasoned that incorporating these measures into the CSS formulation would enhance the score’s sensitivity in defining similar and different clusters. The proportions of cells expressing a marker in a cluster (pct1) compared to the background (pct2), and the average relative expression level compared to the background (avgLogFC), are generated by Seurat in the marker lists and so can be extracted straightforwardly.

To quantify the degree to which a marker is unique to a particular cluster, we define a *marker uniqueness score* inspired by the work of Carson et al. [75] as ${u}_{i}=1-\sqrt{\frac{{L}_{i}}{{L}_{n}}}$, where ${u}_{i}$ is the uniqueness score, ${L}_{i}$ is the number of clusters associated with each marker gene, and ${L}_{n}$ is the total number of clusters in the WT and KO sets combined. Upon incorporating these additional measurements into ${{CSS}}_{{basic}}$, we get an enhanced formulation ${{CSS}}_{{enh}}$ as

$${{CSS}}_{{enh}}=\frac{\mathop{\sum }\nolimits_{i=1}^{{C}_{12}}{w}_{i1}{{\cdot }}{u}_{i}+\mathop{\sum }\nolimits_{i=1}^{{C}_{12}}{w}_{i2}{{\cdot }}{u}_{i}}{\mathop{\sum }\nolimits_{i=1}^{{C}_{12}}{w}_{i1}{{\cdot }}{u}_{i}+\mathop{\sum }\nolimits_{i=1}^{{C}_{12}}{w}_{i2}{{\cdot }}{u}_{i}+\mathop{\sum }\nolimits_{j=1}^{{U}_{1}}{w}_{j1}{{\cdot }}{u}_{j}+\mathop{\sum }\nolimits_{k=1}^{{U}_{2}}{w}_{k2}{{\cdot }}{u}_{k}}$$

where ${C}_{12}$, ${U}_{1}$, and ${U}_{2}$ carry the same meanings as in ${{CSS}}_{{basic}}$. Variables ${u}_{i}$, ${u}_{j}$, and ${u}_{k}$ are the uniqueness scores and ${w}_{i1}$, ${w}_{i2}$, ${w}_{j1}$, and ${w}_{k2}$ are the weights for genes $i$, $j$, and $k$ as indicated in the first subscript and corresponding to the clusters indicated in the second subscript. The weight ${w}_{i1}$ for gene $i$ in cluster 1 is defined as ${{avgLogFC}}_{i1}\left(\frac{{{{pctF}}_{i1}}^{2}}{{{pctB}}_{i1}}\right)$, where ${{avgLogFC}}_{i1}$ is the average log fold change, ${{pctF}}_{i1}$ is the proportion of cells expressing gene $i$ in cluster 1, and ${{pctB}}_{i1}$ is the proportion of cells expressing gene $i$ in the appropriate background population for cluster 1. All other weights, ${w}_{i2}$, ${w}_{j1}$, and ${w}_{k2}$ are defined in the same manner.

Before arriving at the above final formulation, six other variations of this score, including the introduction of only one measurement at a time or combinations thereof, were tested and the results analyzed. In the end, the above formulation, which simultaneously incorporates the uniqueness, strength of expression, and extent of expression (i.e., the percentage of cells that express the gene), proved to be most effective in improving the sensitivity of the score in defining similar and different clusters. A gene that is close to being uniquely expressed, is more strongly expressed, and is more widely expressed is awarded a higher weight than a gene that is shared, weakly expressed, and narrowly expressed. Just like ${{CSS}}_{{basic}}$, ${{CSS}}_{{enh}}$ values range between 0 and 1.

For a given pair of res and nPCs values, if there are $m$ WT clusters and $n$ KO clusters, there would be a total of $m\times n$ ${{CSS}}_{{enh}}$ values. These can be assembled into a structure we call the CSS matrix. The CSS matrix provides a snapshot of the one-to-one relationships between the WT and KO clusters. It lists the pairwise similarity scores, comparing each WT cluster with every KO cluster to quantify how similar or different they are. For example, for our spleen data, the WT-KO dataset pair generated a total of 126 CSS matrices, one for every combination of res and nPCs.

The goal of the second major step of our strategy is to compute a single metric representing each CSS matrix that aims to quantify the overall clustering quality of the res and nPCs parameter value pair associated with the CSS matrix. To achieve this goal, we first compute, for each row and column, a *distinction index* (DI) equal to the difference between the maximum element and the mean of the remaining elements. This yields a total of $m$ row-level and $n$ column-level distinction indices. We express the mean value of this set of $m\times n$ indices as $\tfrac{1}{{mn}}\left[{\sum }_{i=1}^{m}{d}_{{row|i}}+{\sum }_{j=1}^{n}{d}_{{col|j}}\right]$, which we refer to as the distinction index for the entire CSS matrix (CaSSiDI). Thus, CaSSiDI represents a quantification of the clustering quality associated with a given res/nPCs parameter pair choice. Clustering quality can have many different meanings depending on the context. Here, we interpret the DI of a CSS matrix and, by extension, the corresponding res/nPCs parameter pair choice, as a metric that quantifies how well the parameter pair choice has been able to identify matching WT and KO clusters while also identifying and segregating any clusters in either genotype that do not have a good match in the other genotype. For our spleen data, the set of 126 CaSSiDI values for a pair of WT-KO datasets can then be directly used to rank the parameter pair choices from best to worst, with the highest CaSSiDI value corresponding to the best parameter pair choice.

### Nebula plot

In Fig. 3H, for each gene, four violin plots are shown. The inner two violins correspond to the ILR (left) and NPR (right) subsets, while the outer two violins correspond to their respective background populations used by Seurat for marker identification. Black diamonds represent the average expression level used by Seurat. Genes are ordered as follows: from left to right, those labeled in blue are in decreasing order of inner blue violin diamond level, while those labeled in orange are in increasing order of inner orange violin diamond level. Those labeled in gray are ordered based on the mean of the inner blue and orange violin diamond levels, with highest mean at the center and falling off on either side. The bar plots at the top show the proportion of cells in the cluster (ILR or NPR) that expresses the given gene.

Thus, through the Nebula plot, it is possible to get an overview of the most important genes characterizing a given cluster in terms of differential expression compared to background. Moreover, it is possible to compare two clusters at a time and get an idea of how widely each gene is expressed in a population through the bar plots.

### Supplementary information

Supplemental Information

Supplemental Figure 1

Supplemental Figure 2

Supplemental Figure 3

Supplemental Figure 4

Supplemental Figure 5

Supplemental Figure 6

Supplemental Figure 7

Supplemental Figure 8

Table S1

Table S2

Table S3

Table S4

Table S5

Table S6

Table S7

Table S8

Table S9

Table S10

Table S11

Table S12

Table S13

Table S14

Table S15

Table S16

Reporting Checklist

## Data Availability

The 10X single-cell sequencing raw data files and processed matrices associated with this study have been deposited in the Gene Expression Omnibus database under the accession code GSE252466.

## Code availability

A software implementation of CaSSiDI along with critical scripts used to analyze the scRNAseq data presented in the paper can be found at https://github.com/CodeInTheSkies/CaSSiDI-PirB-Macrophage-Paper.
