# Supplementary material for: CaSSiDI: novel single-cell “Cluster Similarity Scoring and Distinction Index” reveals critical functions for PirB and context-dependent Cebpb repression
Source: Cell Death Differ. 2024 Feb 21;31(3):265–79. doi: 10.1038/s41418-024-01268-8 (PMC10923835; doi:10.1038/s41418-024-01268-8)
Supplement: Supplementary file 1 — Supplemental Information [file 41418_2024_1268_MOESM1_ESM.docx]

**Supplemental Information**

**Supplemental Methods**

**B16 tumor tissue processing**

B16 tumor resection, tumor tissue processing, and sorting of MerTK^+^CD64^+^ tumor-associated macrophages (TAMs) were performed as previously described [1]. Single-cell RNA sequencing (scRNAseq) was performed for TAMs isolated from B16 tumor tissues as described in the Main Methods.

**Rationale and derivation of the CaSSiDI method for guiding cell cluster determination in scRNAseq datasets**

When comparing two scRNAseq samples separately in a side-by-side fashion, the ideal number of clusters is the lowest number needed to separate potential cellular activation states and functionalities. Therefore, clusters should be as distinct as possible and, under ideal circumstances, approaches like “overclustering followed by manual merging of redundant clusters” should not be necessary. In addition, the number of clusters of two biological replicates should be the same, and the clusters should be as similar as possible in their marker gene expression.

Applying the widely used combined clustering approach facilitates the clustering and comparison of two or more scRNAseq datasets in a single shot. However, utilizing this approach for comparing wild type (WT) with genetically manipulated cells (e.g., knockout cells) might affect the authentic clustering of the WT cells. We encountered this problem firsthand: *Lifr*-expressing TAMs with a rudimentary monocytic phenotype could only be detected in the PirB KO TAM population when it was analyzed in the presence of WT TAMs (combined analysis). This conundrum emphasizes the importance of a separate side-by-side clustering approach that independently clusters the two samples. Sequential use of both strategies is beneficial because each provides insights from a different angle, facilitating detailed investigation of two scRNAseq datasets. The use of the combined clustering approach alone can also be confounded by discrepancies in transcriptome measurements that correlate with diverse groups of cells in a given type of experiment; for example, comparisons among more than two groups of cells, or among experiments performed at different time points in the same laboratory (which require *batch correction)*, or among different datasets obtained by different laboratories (which requires the extended correction processes of *data integration)*. Thus, any combined analysis is likely affected by technical and biological flaws, and data integration still represents a challenge in the scRNAseq field [2]. No amount of sophisticated data integration is likely to be able to eliminate batch effects and such confounders completely and cleanly from the analysis without affecting or biasing the potential biological insights originally expected from the data.

A major disadvantage of using data integration or batch correction is the possibility of over-correction. No method can perfectly correct only for batch effects and leave all other real-world biological differences intact. That boundary is very elusive, and so all methods are virtually guaranteed to either over-correct or under-correct. In addition, certain datasets are more affected than others, and the extent of correction depends on the dataset itself. Therefore, it is beneficial to analyze WT and genetically modified datasets separately and in combination. Even if a powerful data integration method is employed, a combined-only approach will likely miss some critical biological differences due to over-correction. These differences are most likely to be detected if a separate clustering approach is also performed. CaSSiDI facilitates such an approach and circumvents batch correction and data integration requirements by comparing separate cluster conditions of two scRNAseq datasets.

The design of CaSSiDI takes three primary considerations into account. First, only positive markers (genes overexpressed in a cluster compared to the background population) are included to determine cluster similarities. Although examination of negative markers (genes underexpressed in a cluster compared to the background population) is essential for characterizing a particular cell subset, such genes are often redundant across several clusters, rendering them ambiguous and of a noise-like character. This consideration is analogous to cell lineages in the following way. Suppose we aim to identify B lineage cells in a single cell suspension derived from the spleen. Then, it is more informative to know that B lineage cells do express CD19 than knowing that B lineage cells do not express CD3. In the former scenario, we can identify B lineage cells by utilization of the positive selection marker CD19; in the latter scenario, we would end up purifying B lineage cells, including all other cell lineages found in the single-cell suspension except for cells that express CD3 (mainly T and NKT cells). Therefore, knowing positive markers is more informative, instructive, and exclusive in the identification procedure than incorporating often redundantly assigned negative markers.

Secondly, we designed CaSSiDI so that markers uniquely associated with cells in a given cluster are weighted and scored as particularly influential. This strategy is analogous to the lineage- and subset-identifying genes like CD19, the T cell antigen receptor, or the signature cytokines linked to discretely defined T helper subsets, e.g., IL-4, IL-10, IL-12, and IL-17. Thus, we also considered a homogeneous expression of cluster-associated markers as particularly influential in scoring cell identities and states.

Thirdly, we factored in the level of marker gene expression whenever a given marker gene was expressed in two or more clusters.

Please see the Main Methods for information on the mathematical structure of CaSSiDi.

**Supplemental Results**

In addition to data bolstering the results shown in the Main Figures, the following Supplemental Results (which were obtained during the course of our study) include data of interest that invite future experimentation.

**DEGs in WT NPR vs. WT ILR subsets of splenic red pulp macrophages (RPMs)**

A direct comparison of DEGs between NPRs and ILRs showed upregulation of ribosomal genes in the latter, presumably reflecting elevated protein translation and active PI3K signaling (Fig. S2B, right).

**DEGs in WT vs. PirB KO peritoneal macrophages (PMs)**

The T2M (*Tlr2*-expressing macrophages) cluster was highly similar in the WT and PirB KO PM populations, with only *Cebpb* and *Rps28* being increased in the mutant (Fig. S5B, C, D). T2M cells expressed *Tlr2, Tnf, Icam1, Cxcl2,* *Il6*, which are all needed by LPMs to combat *S. aureus* infections [3, 4]. The T4M cluster (*Timd4*-expressing macrophages) comprised Timd4^+^ LPMs expressing *Cxcl13, Cd63, Wnt2, Marco, Arg1, Saa3, Pf4* (Fig. S5B, C, D). The most pertinent WT vs. PirB KO DEGs in the T4M cluster were *H-2 class II, Lyz1, Pdia3, C1qb, Uba52, Tpt1, Ifi27l2a*, *Cebpb* and ribosome-associated genes (Fig. S5D). The HSM cluster [*Heat stable antigen (CD24)*-expressing macrophages] showed the least similarity between WT and PirB KO samples (Fig. 6B), but both expressed CD24 (Fig. S5B, C, D; Table S9, S10). Additional marker genes expressed by both WT and PirB KO HSM cells included several S100 alarmins, *Serpinb1a* (cytosolic proteinase) [5], and two prostaglandin metabolism-associated genes (*Ptgis*, *Ptgs1*) that are linked to PM maturation and *S. aureus* pathology [6, 7] (Fig. S5B). The WT vs. PirB KO DEGs in the HSM clusters were similar to those in the T4M clusters (Fig. S5C, D).

**DEGs in WT vs. PirB KO tumor-associated macrophages (TAMs)**

Tumor-associated macrophages are the most abundant leukocytes in the tumor microenvironment (TME) of many solid cancers. TAMs emerge during chronic inflammation in the TME and maintain an immunosuppressive, cancer-protective environment [8-11]. Most TAMs promote immune tolerance by suppressing effector T cells and inducing expansion of regulatory T cells (Tregs). PirB-deficient myeloid-derived suppressor cells (MDSCs) reportedly show decreased immunosuppressive potential and instead become immunostimulatory [12].

To investigate *in vivo* the effects of PirB deficiency on TAM heterogeneity and gene expression patterns, we applied the syngeneic B16 melanoma tumor graft model to our WT and PirB KO mice [13, 14]. As expected, tumors grew significantly slower in B16 PirB KO mice than in B16 WT littermates (Fig. S6A). We isolated TAMs co-expressing CD64 and Mertk [1] and used CaSSiDI to determine the optimal clustering condition: a res of 0.4 plus nPCs of 15 (Fig. S6B). Separate clustering revealed 6 TAM subsets in the WT population and 5 TAM subsets among PirB KO cells. Both WT and PirB KO TAMs contained a similar cycling population (CYT) of cells expressing the classical macrophage markers *Mrc1*, *C1qa* *C1qc* (Fig. S6C, D, E, F; Table S11, S12).

One PirB KO cluster (Nlrp3 inflammasome TAMs; NITs) was similar to two WT clusters, namely WT NITs and *Lifr*^+^ monocytic cells (LMOs) (Fig. S6C, D, E, F). Lifr signaling is known to promote monocyte differentiation into macrophages [15] as well as TAM generation in the TME [16]. WT LMOs also expressed *Plac8, Ccr2, Ly6c2*, classifying them as myeloid-derived suppressor cells (MDSCs) or recently established TAMs (Fig. S6D, E, F, H, I; Table S11). In this separate clustering analysis, LMOs were either missing from the PirB KO population, or PirB KO NITs showed a gene expression pattern that partially overlapped that of WT LMOs (Fig. S6C). Another MDSC-like cluster with an ISG signature (IMO) was present in WT and PirB KO TAM populations. IMOs expressed the monocytic markers *Plac8, Ccr2, Ly6c2,* and the ISG-related genes *Ifit1, Ifit2, Ifit3 Cxcl10, Mx1, Oasl1* (S6D, E, F, H, I; Table S11, S12). Interestingly, *Brd2* and *Irf7* mRNAs were decreased in mutant IMOs, but GSVA indicated an upregulation of pathways of complement system activation (Fig. S6J).

We next applied combined clustering to investigate the apparent absence of LMOs among PirB KO TAMs. Notably, this analysis imposed the appearance of a small cluster of KO cells with an LMO-like signature (yellow cluster in Fig. S6 panels). In addition, KO cells from an enlarged NIT cluster occupied the region of the LMO cluster (light-blue cluster in Fig. S6 panels). These changes were also reflected in higher similarities in the CSS table (Fig. S6C). Numerous MDSC- and TAM-relevant DEGs were identified in LMOs (Fig. S6I). *Cebpb* expression was present in all clusters and highest in LMOs, which, in the separate clustering approach, were absent in the PirB KO sample (Fig. S6G). GSVA revealed that WT LMOs showed increased Toll-like receptor signaling compared to the mutant, whereas the LMO-like PirB KO cluster showed enhanced activation of the “Reactome gluconeogenesis” pathway (Fig. S6J).

A combined clustering analysis-based Slingshot trajectory assessment of WT LMOs showed that the differentiation of the least mature WT LMOs bifurcated to give rise to ISG monocytes and NITs (Fig. S6G); however, in the KO sample, LMOs appeared to adopt a NIT state before becoming ISG monocytes, which seems highly improbable. This outcome thus emphasizes the usefulness of the separate clustering analysis.

From the NIT state, WT cells matured into three different TAM subsets, namely, Prdx-expressing TAMs (PETs), coagulation factor F7-expressing TAMs (F7Ts), and TAMs expressing transferrin receptor protein-1 (Tfrc-expressing TAMs; TFTs). In contrast, PirB KO NITs differentiated along two branches. In the first branch, NITs became F7Ts that gave rise to PETs; in the second branch, NITs matured directly into TFTs (Fig. S6G).

Compared to WT F7Ts, PirB KO F7Ts showed markedly decreased *Klf4* and *Pfn1* (positive regulators of macrophage immunosuppressive functions) [17, 18], and reduced *Nrp1* (represses proinflammatory cytokines) [19] (Fig. S6H, I)**.** GSVA revealed that PirB KO F7Ts showed greater activation of complement system-related pathways than WT F7Ts (Fig. S6J).

WT and PirB KO NITs showed comparable expression of *Il1a, Il1b*, *Ccl6, Cxcl3, CD14, Cxcl7* (Fig. S6E, F, H, I; Table S11, S12). As expected, *Il1a*, *Il1b*, *Tnf* levels were higher in the WT cluster, but *Emp1*, which skews cells towards an anti-inflammatory phenotype [20], was also increased in WT TAMs (Fig. S6H, I). GSVA revealed a reduction in “Reactome Ddx58 Ifih1 mediated induction of interferon alpha-beta and Reactome pexophagy” in WT NITs, with increased activation of “Reactome scavenging by class a receptors” and “Reactome chylomicron remodeling” in the mutant (Fig. S6J).

Transferrin receptor protein-1, encoded by *Tfrc*, facilitates iron uptake through endocytosis of iron-loaded transferrin glycoprotein (*Tf*), and this iron has critical functions in solid tumors [21-24]. Both WT and PirB KO samples contained a TFT cluster. The expression of *Lipa*, which promotes the polarization of melanoma-associated macrophages [25], was elevated in PirB KO TFTs (Fig. S6H, I). However, these PirB-deficient TFTs expressed less *Timp2* (Fig. S6H, I), which is needed to promote tumor vascularization [26]. GSVA revealed increased engagement of “Reactome muscarinic acetylcholine receptors” pathways in PirB KO TFTs compared to WT TFTs, as well as downregulation of autophagy-related pathways in WT TFTs (Fig. S6J).

Among PETs, both the WT and PirB KO subsets expressed the inflammatory mediator *Prdx1* and the transcription factor *Bhlhe41* (Fig. S6H, I). Only WT PETs expressed *CD274*, encoding the signaling inhibitor PD-L1 (Table S11, S12). Compared to WT PETs, PirB KO PETs exhibited decreased *Rsrp1* (tissue healing) and *Sertad1* (immunosuppression) but elevated *Adm* (vasorelaxant) (Fig. S6H, I).

Out of curiosity, we examined our WT and PirB KO TAMs for DEGs known to be PirB-dependent and related to the former M1 vs. M2 concepts of TAM phenotypes [27-30]. Although we did find some differential expression, most of the relevant genes (including *Il10, Il4ra, Arg1, Nos2, Ifngr1*) were not among the top DEGs (Fig. S7A-F; Table S11, S12). Notably, *Tnf, Nos2, Arg1, Il10*, all formerly thought to distinguish the M1 and M2 macrophage states, were expressed in a cluster-specific rather than a genotype-specific fashion.

Lastly, prompted by the effect of PirB loss on Cebpb’s role in normal macrophage differentiation, we examined additional functions of this transcription factor in the TAM context. Cebpb promotes immunosuppressive gene expression and anti-inflammatory cytokine production by some macrophage subsets, as well as the generation of MDSCs during sepsis [31-34]. Moreover, PirB deficiency decreases the immunosuppressive capacity of MDSCs [12], while *Cebpb* expression in MDSCs was shown to be essential for their immunosuppressive functions [35], suggesting that PirB-mediated *Cebpb* expression is central in rendering MDSCs immunosuppressive. We did not examine MDSCs in depth in our study and therefore do not know if *Cebpb* is elevated in these cells, but WT and PirB KO TAM populations as a whole did not differ in their *Cebpb* mRNA levels (Fig. S6G). Thus, any immunosuppression exerted by PirB KO TAMs is not due to *Cebpb* upregulation. It is conceivable that enhanced *Cebpb* expression in PirB-deficient MDSCs arriving at a tumor site is sufficient to establish a strongly immunosuppressive TME; however, it could also skew the differentiation of emerging TAMs towards immunosuppressive phenotypes. Additional work is needed to explore the impact of PirB loss on TAM differentiation and functions *in vivo.* Nevertheless, we can say with confidence that our results establish that PirB has a previously underappreciated influence on gene expression patterns in TAMs.

**Performance evaluation of CaSSiDI compared to the Standard Seurat Pipeline (SSP): RPM scRNAseq dataset**

The SSP-associated elbow plot of our RPM scRNAseq dataset suggests an optimal nPC value of nPC=10 (Fig. S8A). Regarding cluster identities, SSP-generated WT splenic macrophage subsets comprised an ILR and an ILR-like cluster that exhibited similar gene expression signatures, including *Csf1r, Ccr3, Trf* (Table S13). CaSSiDI also assigned these marker genes to an ILR cluster. However, the SSP ILR-like cluster showed only 21 positively expressed genes as compared to 98 in the SSP ILR cluster, suggesting that these ILR-like cells were ILR progenitors. A similar scenario was observed for the SSP NPR and TRN clusters, each of which was accompanied by another cluster sharing a similar gene expression pattern (NPR-like and TRN-like). Dependencies and trajectories would have to be assessed in additional analyses. However, the NPR and NPR-like gene expression patterns appear so similar that it is difficult to discern why they emerged as separate clusters in the SSP analysis (Fig. S8B).

Another discrepancy can be found in the SSP analyses of *Mertk* and *Spic* expression. The pan-macrophage marker *Mertk* is found only in the SSP ILR and NPR-like clusters, whereas the splenic macrophage marker *Spic* is confined to SSP NPR and NPR-like cells. However, in bona fide splenic red pulp macrophages, *Mertk* and *Spic* are co-expressed, which makes the SSP-generated optimal clustering condition dubious. In addition, the SSP TRN2 cluster contains only 12 positively regulated genes that are exclusively mitochondrial (Table S13). Thus, the subdivision of ILRs, NPRs, and TLRs to generate ILR-like, NPR-like, and TLR-like clusters, plus the lack of separate follicular (FOB) and marginal zone (MZB) B cell clusters, makes the SSP-generated clustering outcome harder to interpret and less accurate than the optimal clustering condition determined by CaSSiDI.

SSP generated a similar clustering outcome for the PirB KO splenic macrophage dataset, producing ILR, ILR-like, NPR, and NPR-like subsets but only one TRN cluster. SSP also generated two MZB clusters (MZB and MZB2) with high expression of the MZB marker gene *Mzb1*, but no FOB cluster was identified (Fig. S8B; Table S14). In addition, we found that the MZB2 cluster showed increased expression of CD79a, which is a direct target gene of the transcription factor Ebf1 and dependent on Ebf1 expression. However, Ebf1 was not on the list of MZB2-associated positively regulated genes. This outcome supports our contention that SSP clustering conditions in this context are suboptimal, distorted, and/or artificial.

**Performance evaluation of CaSSiDI compared to SSP: PM scRNAseq dataset**

The SSP-associated elbow plot suggests an optimal nPC value of nPC=10 (Fig. S8C), but obvious defects were observed in our SSP analysis of our WT and PirB KO peritoneal macrophage datasets. The SSP WT analysis yielded three different INT clusters (INT, INT2, INT3), with INT2 and INT3 containing only 2 or 1 positively expressed marker genes, respectively (Fig. S8D; Table S15). This outcome appears highly artificial, and it is hard to discern why those three clusters should exist separately. In addition, the SSP WT sample lacked a T2M cluster, and its key markers *Tlr2* and *Il6* were absent from the entire marker gene list, although this cluster was identified using the same clustering condition in the SSP PirB KO sample. CaSSiDI, however, identified a robust T2M cluster in both samples. For the PirB KO sample, SSP analysis generated two T4M clusters with a similar gene expression signature and several shared marker genes, including *Marco, Fscn1, Arg1, Wnt2, CD63*. The SSP INT, INT2, and INT3 clusters were also generated for the PirB KO sample, with INT2 cells expressing a few genes (*Bcam, H2-DMa, Tmem176a, Lyz1*) that are typical for an SPM-related gene expression signature (Fig. S8D; Table S16).

In our opinion, our examination of SSP-generated clustering conditions applied to our data indicate that SSP constitutes an outdated approach characterized by “over-clustering followed by manual merging of redundant clusters”. In contrast, our CaSSiDI method delivers enhanced and more biologically accurate clustering outcomes.

**Supplementary Figure Legends**

**Figure S1. Steady-state monocytes: CaSSiDI reveals plausible WT and PirB KO clusters (related to Figure 2).**

**A.** Bubble plots showing prominent monocyte-related DEGs (PirB-dependent in black; cluster-specific in red) between the TRN and ISG clusters derived from WT and PirB KO monocytes. **B-E.** Volcano plots showing markers (left) and DEGs (right) revealed by comparing the indicated clusters of WT vs. PirB KO monocytes. **F.** Top differentially expressed pathways identified by GSVA between the TRN and ISG clusters derived from WT and PirB KO monocytes. Also shown are t-value scores from a linear model analysis conducted on GSVA scores. **G.** Elbow plot showing the correlation of the indicated parameters for the monocyte scRNA-seq data. **H.** UMAPs showing SSP-based separately clustered WT vs. PirB KO monocytes.

**Figure S2. Spleen: WT NPR vs. WT ILR clusters (related to Figure 3).**

**A.** Top five parameter value pair choices as determined by CaSSiDI (*Res*: clustering resolution, *nPCs*: number of principal components). **B.** Volcano plots showing markers (left) and DEGs (right) revealed by comparing WT NPRs vs. WT ILRs. **C.** Histograms showing flow cytometric assessment of expression levels of the indicated cell surface markers in WT RPMs. **D.** Gene expression levels (relative to actin) of the indicated genes as determined by qPCR. *P<0.05, **P<0.01 as determined by regression analysis with two-way analysis of variance (ANOVA); each comparison stands alone (Fisher’s LSD test). Data are from one experiment assessing technical duplicates of three biological replicates.

**Figure S3. Spleen: WT NPR and ILR clusters vs. PirB KO** **NPR and ILR clusters (related to Figure 4).**

**A.** UMAPs showing separately clustered WT vs. PirB KO splenic RPMs. **B.** CSS table comparing WT vs. PirB KO RPMs. **C.** Volcano plots showing markers (left) and DEGs (right) revealed by comparing WT vs. PirB KO ILRs (top) and NPRs (bottom). **D.** Top differentially expressed pathways identified by GSVA in WT vs. PirB KO NPRs (top) and ILRs (bottom). Also shown are t-value scores from a linear model analysis conducted on GSVA scores.

**Figure S4. Validation of CaSSiDI utilizing scRNA-seq data from Zeb2 KO RPMs (related to Figure 5).**

**A.** UMAPs showing separately clustered spleen cells from our WT mice (data from this study) compared with spleen cells from Zeb2 WT and Zeb2 KO mice (data from the study by Scott; [36]). Colors are matched to show same or similar cell types, as determined by CaSSiDI. **B.** CSS table comparing WT clusters from our data (PirB WT) with WT clusters from the Scott data (Zeb2 WT). **C.** Top five parameter value pair choices as determined by CaSSiDI (*Res*: clustering resolution, *nPCs*: number of principal components). The top choice of res=0.4, nPCs=35 was used as outlined in the Main Results section. **D.** Volcano plots showing markers (left) and DEGs (right) revealed by comparing the indicated clusters from WT and Zeb2 KO mice.

**Figure S5. Peritoneum: WT vs. PirB KO peritoneal macrophages (related to Figure 6).**

**A.** Left: Flow cytometric gating strategy for the identification of (top) CD45.1^+^ recipient LPMs and (bottom) donor CD45.2^+^ LPMs from either WT or PirB KO mice. Right: Quantitation of WT and PirB KO LPMs recovered from CD45.1^+^ recipients at day 21 after co-transplantation (WT n=4, KO n=3). **B, C.** Volcano plots showing (**B**) markers and (**C**) DEGs revealed by comparing the indicated clusters of WT and PirB KO PMs. **D.** Bubble plots showing prominent DEGs between the indicated clusters of WT and PirB KO PMs.

**Figure S6. B16 melanoma model: WT vs. PirB KO mice.**

**A.** Quantitation of B16 tumor growth following subcutaneous transplantation into WT (n=5) and PirB KO (n=6) female littermate recipients. *P<0.05, **P<0.01 as determined by regression analysis with two-way ANOVA followed by Sidak’s post hoc multiple comparison test. Data are representative of one experiment. **B.** Top five parameter value pair choices as determined by CaSSiDI. The top choice of res=0.4, nPCs=15 was used to cluster the WT and PirB KO TAM datasets. **C.** CSS table corresponding to the top choice in **B**. **D.** UMAPs of separately clustered WT and PirB KO TAM populations. **E, F.** Prominent marker genes for the indicated (by color) clusters derived from WT (**E**) and PirB KO (**F**) TAMs. **G.** UMAPs showing combined clustering of WT and PirB KO TAMs, with cells grouped by genotype, by separate-clustering identities superimposed with slingshot trajectory curves originating from the *LMO* cluster, and by *Cebpb* expression level. **H.** Ridge plots of selected DEGs between the indicated clusters of WT and PirB KO TAMs. **I.** Bubble plot showing prominent DEGs between the indicated clusters of WT and PirB KO TAMs. **J.** Top differentially expressed pathways identified by GSVA of the indicated clusters derived from WT and PirB KO TAMs. Also shown are t-value scores from a linear model analysis conducted on GSVA scores.

**Figure S7. B16 melanoma: PirB-dependent genes related to the M1 vs. M2 concept.**

**A-F.** Bubble plots showing prominent DEGs between the indicated clusters derived from WT and PirB KO TAMs.

**Figure S8. CaSSiDI vs. Standard Seurat Pipeline (SSP).**

**A.** Elbow plot showing the correlation of the indicated parameters for the splenic macrophage scRNA-seq data. **B.** UMAPs showing SSP-based separately clustered WT vs. PirB KO splenic RPMs. **C.** Elbow plot showing the correlation of the indicated parameters for the peritoneal macrophage (PM) scRNA-seq data. **D.** UMAPs showing SSP-based separately clustered WT vs. PirB KO PMs.

**Supplemental References**

1. Kubli SP, Vornholz L, Duncan G, Zhou W, Ramachandran P, Fortin J, Cox M, Han S, Nechanitzky R, Nechanitzky D, et al: **Fcmr regulates mononuclear phagocyte control of anti-tumor immunity.** *Nat Commun* 2019, **10:**2678.

2. Luecken MD, Theis FJ: **Current best practices in single-cell RNA-seq analysis: a tutorial.** *Mol Syst Biol* 2019, **15:**e8746.

3. Bishayi B, Bandyopadhyay D, Majhi A, Adhikary R: **Possible role of Toll-like receptor-2 in the intracellular survival of Staphylococcus aureus in murine peritoneal macrophages: involvement of cytokines and anti-oxidant enzymes.** *Scand J Immunol* 2014, **80:**127-143.

4. Jorch SK, Surewaard BG, Hossain M, Peiseler M, Deppermann C, Deng J, Bogoslowski A, van der Wal F, Omri A, Hickey MJ, Kubes P: **Peritoneal GATA6+ macrophages function as a portal for Staphylococcus aureus dissemination.** *J Clin Invest* 2019, **129:**4643-4656.

5. Burgener SS, Leborgne NGF, Snipas SJ, Salvesen GS, Bird PI, Benarafa C: **Cathepsin G Inhibition by Serpinb1 and Serpinb6 Prevents Programmed Necrosis in Neutrophils and Monocytes and Reduces GSDMD-Driven Inflammation.** *Cell Rep* 2019, **27:**3646-3656 e3645.

6. Zaslona Z, Serezani CH, Okunishi K, Aronoff DM, Peters-Golden M: **Prostaglandin E2 restrains macrophage maturation via E prostanoid receptor 2/protein kinase A signaling.** *Blood* 2012, **119:**2358-2367.

7. Wu J, Liu B, Mao W, Feng S, Yao Y, Bai F, Shen Y, Guleng A, Jirigala B, Cao J: **Prostaglandin E2 Regulates Activation of Mouse Peritoneal Macrophages by Staphylococcus aureus through Toll-Like Receptor 2, Toll-Like Receptor 4, and NLRP3 Inflammasome Signaling.** *J Innate Immun* 2020, **12:**154-169.

8. Hegde S, Leader AM, Merad M: **MDSC: Markers, development, states, and unaddressed complexity.** *Immunity* 2021, **54:**875-884.

9. Grover A, Sanseviero E, Timosenko E, Gabrilovich DI: **Myeloid-Derived Suppressor Cells: A Propitious Road to Clinic.** *Cancer Discov* 2021, **11:**2693-2706.

10. Tesi RJ: **MDSC; the Most Important Cell You Have Never Heard Of.** *Trends Pharmacol Sci* 2019, **40:**4-7.

11. Binnewies M, Roberts EW, Kersten K, Chan V, Fearon DF, Merad M, Coussens LM, Gabrilovich DI, Ostrand-Rosenberg S, Hedrick CC, et al: **Understanding the tumor immune microenvironment (TIME) for effective therapy.** *Nat Med* 2018, **24:**541-550.

12. Ma G, Pan PY, Eisenstein S, Divino CM, Lowell CA, Takai T, Chen SH: **Paired immunoglobin-like receptor-B regulates the suppressive function and fate of myeloid-derived suppressor cells.** *Immunity* 2011, **34:**385-395.

13. Qian BZ, Pollard JW: **Macrophage diversity enhances tumor progression and metastasis.** *Cell* 2010, **141:**39-51.

14. Halaby MJ, Hezaveh K, Lamorte S, Ciudad MT, Kloetgen A, MacLeod BL, Guo M, Chakravarthy A, Medina TDS, Ugel S, et al: **GCN2 drives macrophage and MDSC function and immunosuppression in the tumor microenvironment.** *Sci Immunol* 2019, **4**.

15. Meisel SR, Shimon I, Edgington TS, Melmed S, Cercek B, Shah PK: **Leukaemia inhibitory factor enhances tissue factor expression in human monocyte-derived macrophages: a gp130-mediated mechanism.** *Br J Haematol* 1999, **107:**747-755.

16. Duluc D, Delneste Y, Tan F, Moles MP, Grimaud L, Lenoir J, Preisser L, Anegon I, Catala L, Ifrah N, et al: **Tumor-associated leukemia inhibitory factor and IL-6 skew monocyte differentiation into tumor-associated macrophage-like cells.** *Blood* 2007, **110:**4319-4330.

17. Liao X, Sharma N, Kapadia F, Zhou G, Lu Y, Hong H, Paruchuri K, Mahabeleshwar GH, Dalmas E, Venteclef N, et al: **Kruppel-like factor 4 regulates macrophage polarization.** *J Clin Invest* 2011, **121:**2736-2749.

18. Zhang J, Li S, Zhang X, Li C, Zhang J, Zhou W: **LncRNA HLA-F-AS1 promotes colorectal cancer metastasis by inducing PFN1 in colorectal cancer-derived extracellular vesicles and mediating macrophage polarization.** *Cancer Gene Ther* 2021, **28:**1269-1284.

19. Dai X, Okon I, Liu Z, Wu Y, Zhu H, Song P, Zou MH: **A novel role for myeloid cell-specific neuropilin 1 in mitigating sepsis.** *FASEB J* 2017, **31:**2881-2892.

20. Lin B, Zhang T, Ye X, Yang H: **High expression of EMP1 predicts a poor prognosis and correlates with immune infiltrates in bladder urothelial carcinoma.** *Oncol Lett* 2020, **20:**2840-2854.

21. DeRosa A, Leftin A: **The Iron Curtain: Macrophages at the Interface of Systemic and Microenvironmental Iron Metabolism and Immune Response in Cancer.** *Front Immunol* 2021, **12:**614294.

22. Liang W, Ferrara N: **Iron Metabolism in the Tumor Microenvironment: Contributions of Innate Immune Cells.** *Front Immunol* 2020, **11:**626812.

23. Sacco A, Battaglia AM, Botta C, Aversa I, Mancuso S, Costanzo F, Biamonte F: **Iron Metabolism in the Tumor Microenvironment-Implications for Anti-Cancer Immune Response.** *Cells* 2021, **10**.

24. Shibabaw T, Teferi B, Molla MD, Ayelign B: **Inflammation Mediated Hepcidin-Ferroportin Pathway and Its Therapeutic Window in Breast Cancer.** *Breast Cancer (Dove Med Press)* 2020, **12:**165-180.

25. Gerloff D, Lutzkendorf J, Moritz RKC, Wersig T, Mader K, Muller LP, Sunderkotter C: **Melanoma-Derived Exosomal miR-125b-5p Educates Tumor Associated Macrophages (TAMs) by Targeting Lysosomal Acid Lipase A (LIPA).** *Cancers (Basel)* 2020, **12**.

26. Guedez L, Jensen-Taubman S, Bourboulia D, Kwityn CJ, Wei B, Caterina J, Stetler-Stevenson WG: **TIMP-2 targets tumor-associated myeloid suppressor cells with effects in cancer immune dysfunction and angiogenesis.** *J Immunother* 2012, **35:**502-512.

27. Orecchioni M, Ghosheh Y, Pramod AB, Ley K: **Macrophage Polarization: Different Gene Signatures in M1(LPS+) vs. Classically and M2(LPS-) vs. Alternatively Activated Macrophages.** *Front Immunol* 2019, **10:**1084.

28. Liu J, Geng X, Hou J, Wu G: **New insights into M1/M2 macrophages: key modulators in cancer progression.** *Cancer Cell Int* 2021, **21:**389.

29. Xue J, Schmidt SV, Sander J, Draffehn A, Krebs W, Quester I, De Nardo D, Gohel TD, Emde M, Schmidleithner L, et al: **Transcriptome-based network analysis reveals a spectrum model of human macrophage activation.** *Immunity* 2014, **40:**274-288.

30. Schultze JL, Schmidt SV: **Molecular features of macrophage activation.** *Semin Immunol* 2015, **27:**416-423.

31. McPeak MB, Youssef D, Williams DA, Pritchett CL, Yao ZQ, McCall CE, El Gazzar M: **Frontline Science: Myeloid cell-specific deletion of Cebpb decreases sepsis-induced immunosuppression in mice.** *J Leukoc Biol* 2017, **102:**191-200.

32. Lamkin DM, Srivastava S, Bradshaw KP, Betz JE, Muy KB, Wiese AM, Yee SK, Waggoner RM, Arevalo JMG, Yoon AJ, et al: **C/EBPbeta regulates the M2 transcriptome in beta-adrenergic-stimulated macrophages.** *Brain Behav Immun* 2019, **80:**839-848.

33. Ruffell D, Mourkioti F, Gambardella A, Kirstetter P, Lopez RG, Rosenthal N, Nerlov C: **A CREB-C/EBPbeta cascade induces M2 macrophage-specific gene expression and promotes muscle injury repair.** *Proc Natl Acad Sci U S A* 2009, **106:**17475-17480.

34. Zhou Q, Xian M, Xiang S, Xiang D, Shao X, Wang J, Cao J, Yang X, Yang B, Ying M, He Q: **All-Trans Retinoic Acid Prevents Osteosarcoma Metastasis by Inhibiting M2 Polarization of Tumor-Associated Macrophages.** *Cancer Immunol Res* 2017, **5:**547-559.

35. Marigo I, Bosio E, Solito S, Mesa C, Fernandez A, Dolcetti L, Ugel S, Sonda N, Bicciato S, Falisi E, et al: **Tumor-induced tolerance and immune suppression depend on the C/EBPbeta transcription factor.** *Immunity* 2010, **32:**790-802.

36. Scott CL, T'Jonck W, Martens L, Todorov H, Sichien D, Soen B, Bonnardel J, De Prijck S, Vandamme N, Cannoodt R, et al: **The Transcription Factor ZEB2 Is Required to Maintain the Tissue-Specific Identities of Macrophages.** *Immunity* 2018, **49:**312-325 e315.
