# Supplementary figures and images for: CaSSiDI: novel single-cell “Cluster Similarity Scoring and Distinction Index” reveals critical functions for PirB and context-dependent Cebpb repression
Source: Cell Death Differ. 2024 Feb 21;31(3):265–79. doi: 10.1038/s41418-024-01268-8 (PMC10923835; doi:10.1038/s41418-024-01268-8)

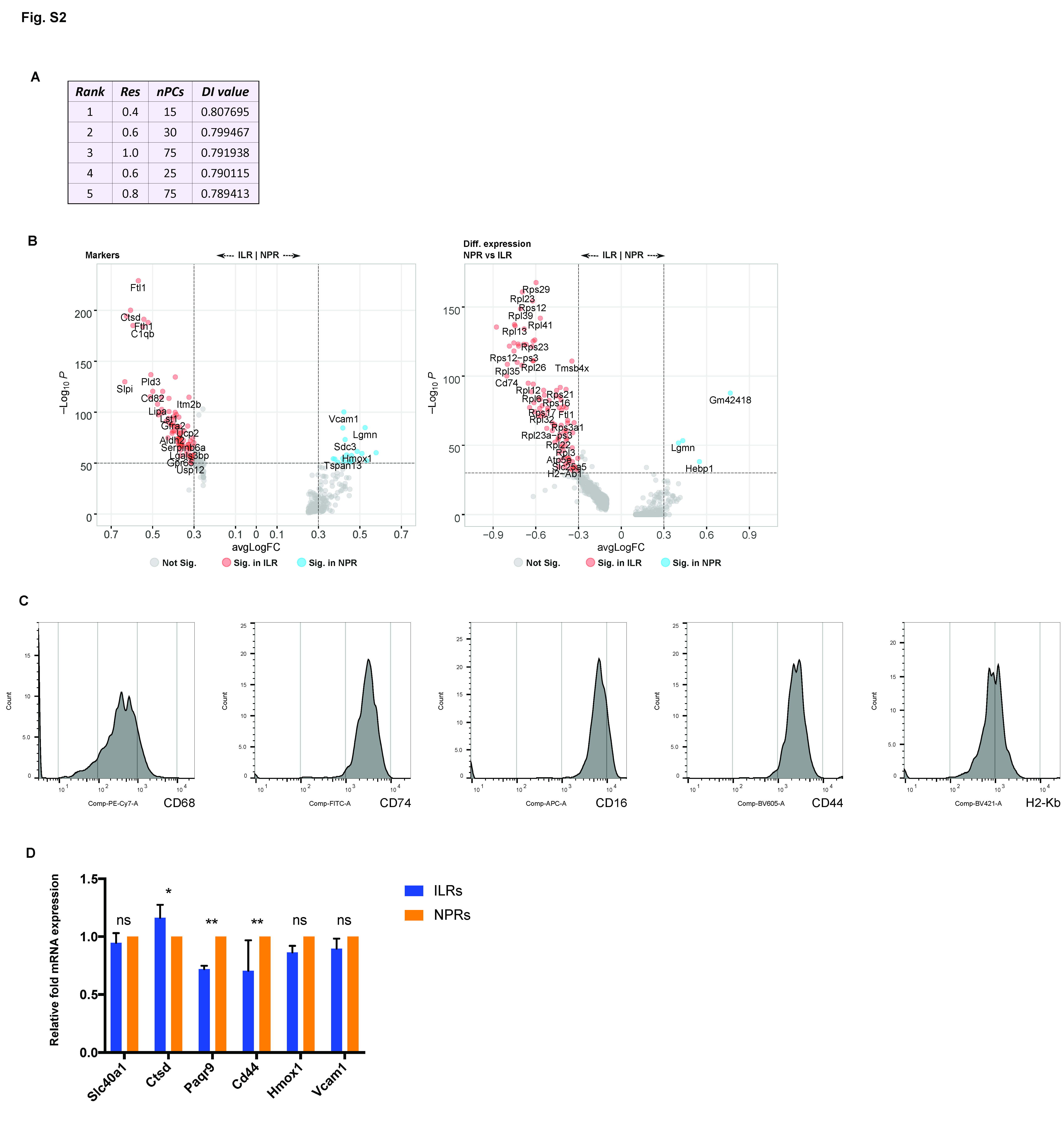

Supplement: Supplementary file 3 — Supplemental Figure 2 [file 41418_2024_1268_MOESM3_ESM.tif]

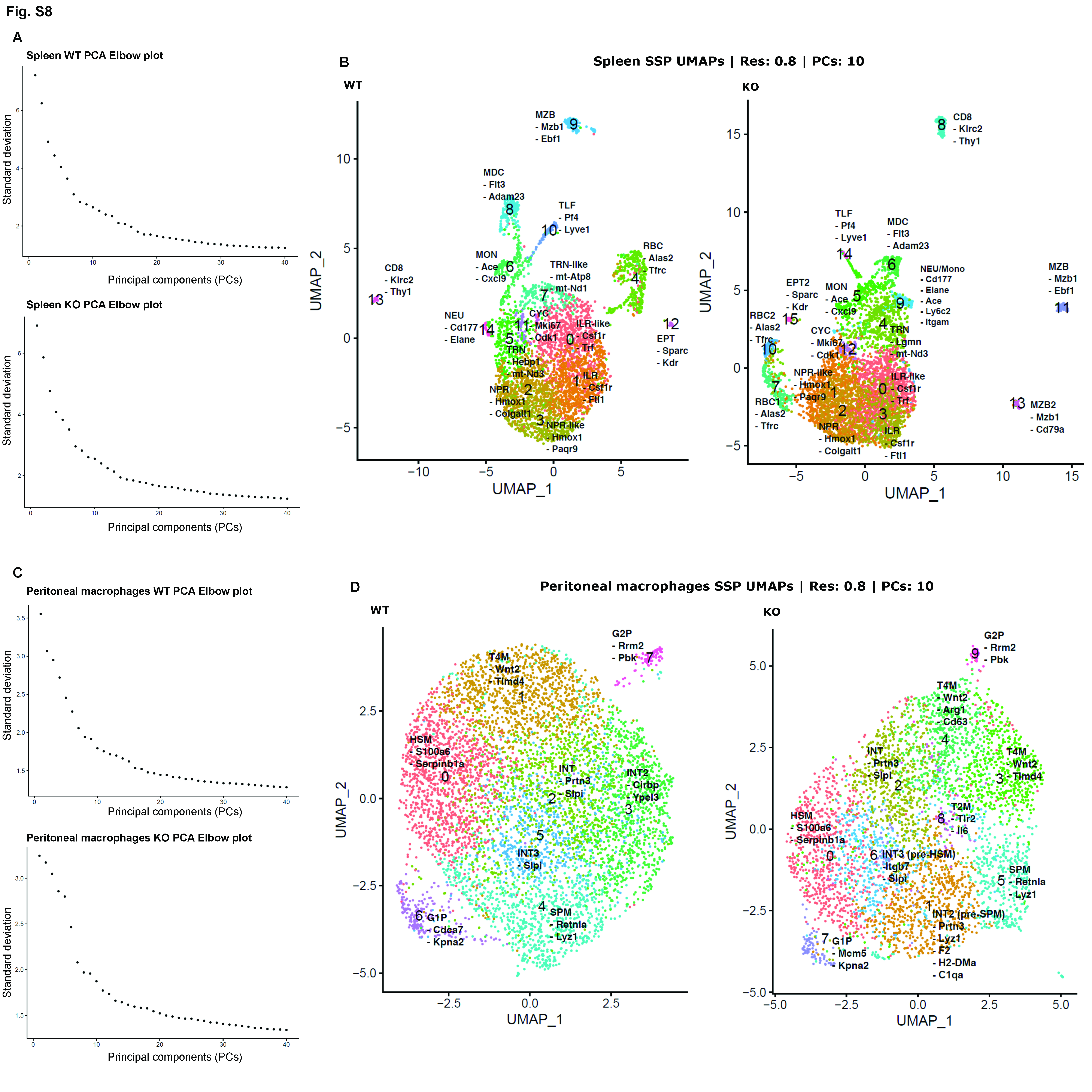

Supplement: Supplementary file 9 — Supplemental Figure 8 [file 41418_2024_1268_MOESM9_ESM.tif]
